# Incremental efficacy systematic review and meta-analysis of psilocybin-for-depression RCTs

**DOI:** 10.1007/s00213-025-06788-w

**Published:** 2025-04-23

**Authors:** Nicholas C. Borgogna, Tyler Owen, Dan Petrovitch, Jacob Vaughn, David A. L. Johnson, Louis A. Pagano, Stephen L. Aita, Benjamin D. Hill

**Affiliations:** 1 Department of Psychological Sciences, Texas Tech University, Lubbock, TX USA; 2 Department of Psychology, University of Alabama at Birmingham, Birmingham, AL USA; 3 St. Cloud VA Health Care Systems, St. Cloud, MN USA; 4 VA Maine Healthcare System, Augusta, ME USA; 5 Department of Psychology, University of Maine, Orono, ME USA; 6 Alabama Department of Psychology, University of South, Mobile, AL USA

**Keywords:** Depression, Psychedelics, Psilocybin, Randomized Controlled Trial, Harm Reporting, Risk of Bias, Effect Size, Mechanisms of Action

## Abstract

**Rationale:**

Psilocybin is a potentially paradigm-shifting depression intervention. We conducted a systematic review and meta-analysis of psilocybin-for-depression randomized controlled trials (RCTs).

**Objectives:**

Systematically assess harm reporting, risk of bias, action mechanism specification, and incremental therapeutic effect sizes in the psilocybin-for-depression RCT literature.

**Methods:**

Assessed databases included PsycINFO, CINAHL, Embase, Medline, Web of Science, and Scopus. Search terms “Psilocybin” or “Psychedelic” were paired with “Depression”, and "Randomized Controlled Trial" or “RCT”.

**Results:**

We identified *k* = 9 RCTs (*k* = 10 subgroups) involving *n* = 602 participants (56% psilocybin). Five studies had low/very low harm quality reporting, opposed to two with high. Most studies demonstrated a high risk of bias. Therapeutic mechanisms of action (MoAs) were discussed in varying detail but rarely assessed in original publications. Psilocybin was moderately superior to controls at reducing depression (*g* = 0.62; 95% CI = 0.27, 0.98). Effects were heterogenous (τ = .47). Smaller studies evidenced stronger effects that favored psilocybin (Egger’s *b*0 = 3.63, *p* = .014). Almost all studies documented financial conflicts of interests.

**Conclusion:**

Psilocybin demonstrates significant depression reduction relative to controls. However, researchers, clinicians, and stakeholders should consider several contextual factors. Effects were moderate and attenuated in larger and better-controlled studies. Harms reporting and risk of bias was high, though partly driven by unique challenges of psilocybin research. MoAs were variably specified but rarely assessed; suggesting it is unclear how depression is reduced. We advise researchers conduct RCTs with active control conditions, larger samples, and include MoA assessments. Independent RCTs from researchers without financial conflicts of interest are needed.

**Supplementary Information:**

The online version contains supplementary material available at 10.1007/s00213-025-06788-w.

Depression affects an estimated 280 million individuals worldwide (World Health Organization [WHO], 2023). Despite decades of research and billions of dollars in funding, debate remains about the underlying etiology and best approach to treatment (Cai et al. 2020; Z. Li et al. 2021). Currently, the latent disease model is the established framework for diagnosing major depressive disorder (MDD) and related depressive disorders. All depression presentations are conceptualized as syndromes “characterized by a clinically significant disturbance in an individual’s cognition, emotion regulation, or behavior that reflects a dysfunction in the psychological, biological, or developmental processes underlying mental functioning” (American Psychiatric Association 2022). We use the broad term “depression” to describe the latent diseases cataloged in the Diagnostic and Statistical Manual of Mental Disorders, Fifth Edition, Text Revision (DSM-5-TR; American Psychiatric Association 2022) and International Classification of Diseases-11 (ICD-11; WHO, 2022). Depression typically involves prolonged sadness or anhedonia as core criteria along with other symptoms, such as changes in sleep or weight. However, others argue that depression has many features determined by individual and cultural differences (Juhasz et al. 2012).

Treating depression is an active area of clinical science. In the United States, the Food and Drug Administration (FDA) currently has approved 30 agents for depression treatment. These act on various neurotransmitter systems, with many affecting the serotonin system. Many non-FDA approved agents are also employed to treat depression (e.g., quetiapine; Ignácio et al. 2018), especially when FDA approved agents fail (Pappa et al. 2024; Reid et al. 2013). Multiple evidence-based behavioral interventions also exist (Butler et al. 2006; Feijo De Mello et al. 2005; Hayes et al. 2012).

While many interventions hold therapeutic potential, there are drawbacks. For example, psychotherapy interventions take a significant amount of time, are often expensive, and finding qualified therapists can be difficult. Similarly, pharmaceuticals, while relatively accessible, are often met with treatment resistance and/or aversive side effects (Cipriani et al. 2018; McIntyre et al. 2023). The FDA approved antidepressants represent disparate pharmacological classes (e.g., esketamine and fluoxetine are both antidepressants yet pharmacologically very different), suggesting that neurobiological mechanisms underlying depression for any given person might differ drastically. Moreover, many clinicians initially take a “guess-and-check” approach with depression prescriptions (Zeier et al. 2018), as the optimal intervention for a given patient is often unclear. Recent research has also cast doubt on the validity of traditional neurotransmitter models, most notably the serotonin theory that underlies many traditional antidepressant interventions (Moncrieff et al. 2023). Some researchers have gone so far as to suggest that the true therapeutic mechanism underlying depression interventions is placebo (Cuijpers & Cristea 2015).

The problems associated with traditional interventions have led researchers to consider alternative frameworks. The *psychedelic renaissance* (Rhee et al. 2023; Schenberg 2018) represents one such paradigm shift. Prominent examples include classic psychedelics, such as lysergic acid diethylamide (LSD) and psilocybin that primarily affect serotonergic systems, and non-classic hallucinogens such as ketamine and 3,4- methylenedioxymethamphetamine (MDMA). Psychedelics occur as natural by-products of various organisms (e.g., mushrooms), but also can be synthetically derived (e.g., LSD). Various psychedelic compounds have been used for millennia as part of spiritual/religious ceremonies and for recreation. In Western science (Swanson 2018), psychedelics were first investigated by late 19th-century (Heffter 1898; Lewin 1888) and mid-twentieth century scholars (Eisner & Cohen 1958; Osmond 1957; Savage & Mccabe 1973). However, by the 1970s, most psychedelic research was halted based on a confluence of media, political, and legal factors (Hall 2022). The past 20 years have been associated with decreasing stigmatization and increasing interest in potential psychedelic health benefits.

Psilocybin has received attention as a potential antidepressant (dos Santos et al. 2021; N. X. Li et al. 2022). Psilocybin is primarily a 5-HT_1A/2A/2C_ agonist, with action on 5-HT2A explaining the well-documented hallucinogenic effects (Kometer et al. 2013; Nichols 2004). Initial modern clinical trials have demonstrated interest for psilocybin as a possible depression intervention (Carhart-Harris et al. 2016; Grob et al. 2011), particularly for those suffering from treatment resistant depression (Carhart-Harris et al. 2016). More recent randomized controlled trials (RCTs) have also demonstrated hopeful results (Goodwin et al. 2022; von Rotz et al. 2023). In 2018, the FDA granted “breakthrough” status for psilocybin as an intervention for treatment resistant depression, and for MDD in 2019. Such results have led to widespread public interest and media coverage (Lamotte 2022). Scholarly opinion reports have also increased, suggesting a therapeutic potential for psilocybin across a diverse range of problems, including minority stress (Ortiz et al. 2022), dementia (Haniff et al. 2024), and compulsive sexual behavior disorder (Wizła et al. 2022).

While interest is high, several scholars have also voiced warnings that the therapeutic benefits of psychedelics (Yaden et al. 2022), including psilocybin (Rucker 2023), are highly preliminary, susceptible to blind penetration/expectancy effects, and that accompanying psychotherapy may drive the therapeutic effects (Meling et al. 2024; van Elk & Fried 2023). In a similar vein, psilocybin researchers have yet to identify definitive *therapeutic* action mechanisms between psilocybin and depression remission. Most psilocybin scholars have either not addressed therapeutic mechanisms (Gukasyan et al. 2022), posited mechanisms without assessing them (Grob et al. 2011), or acknowledged the therapeutic mechanisms are unknown (Johnson & Griffiths 2017). Indeed, the unique subjective effects produced by psilocybin render it challenging to blind clinical trials using traditional methods (Muthukumaraswamy et al. 2021; Nayak et al. 2023). Moreover, the accompanying hallucinations as well as cognitive, emotional, and self-referential changes, also create pause regarding the functionality of widescale intervention implementation (Preller & Vollenweider 2016; Strickland & Johnson 2022).

Given the increased interest, coupled with the noted concerns, a critical review and meta-analysis of extant psilocybin-for-depression RCTs (i.e., psilocybin vs control) is warranted. To date, several meta-analyses have already evaluated the therapeutic effects of psilocybin on depression (Goldberg et al. 2020; Haikazian et al. 2023; N. X. Li et al. 2022; Metaxa & Clarke 2024; Perez et al. 2023; Yu et al. 2022). Many of these were conducted when the literature was notably smaller (e.g., Goldberg et al. 2020). Extant meta-analyses also tend to report net therapeutic effects relative to a statistical null (i.e., testing whether psilocybin intervention reduced depression score relative to baseline). However, almost none of these meta-analyses considered *incremental* therapeutic effects in relation to control interventions. Even Yu et al. (2022), one of the few meta-analyses to examine standard mean differences comparing psilocybin intervention to a control, only included four RCTs in their analyses (as this was the size of the psilocybin RCT literature at the time). Additionally, at least one recent meta-analysis (Metaxa and Clarke 2024) was criticized within days of release due to questionable reporting (Cristea et al. 2024). Given the relatively fast paced nature of psychedelic research, we believe it is important to re-review many of the formerly reviewed RCTs, in addition to the new studies, with a specific aim for incremental efficacy.

Concurrently, popular opinion pieces often describe the benefits of psilocybin with minimized consideration for potential harm. A recent meta-analysis demonstrated that researchers have broadly mischaracterized or underreported potential harms across published ketamine (a similar hallucinogenic agent) for depression trials (Taillefer de Laportalière et al. 2023). The current psilocybin enthusiasm described in popular media could be misleading regarding the risk/benefit trade-offs associated with psilocybin. Indeed, De Giorgi and Ede (2024) noted the contradictory messages pervasive within the psychedelic renaissance, such as psilocybin having “negligible side effects” including “confusional states, substance misuse, intentional self-harm, suicidal behaviour, and psychotic symptoms” (pg. 1). Having estimates of bias risk and harm quality reporting would help stakeholders contextualize any observed incremental therapeutic effects.

Additionally, we were curious of the degree to which psilocybin scholars and practitioners specify and/or measure potential therapeutic mechanisms of action (MoA) within their RCTs. This is common practice in other areas of medicine, but relatively underutilized in psychiatry (e.g., confirming serotonin dysregulation before SSRI prescription). For example, psilocybin is associated with many well documented pharmacological processes, such as 5-HT_1A/2A/2C_ agonist activity (Kometer et al. 2013; Nichols 2004). However, the degree to which such processes are measured and/or explained is less clear. As such, we wanted to synthesize how hypothesized MoAs are considered and/or measured by researchers conducting psilocybin-for-depression RCTs.

## Current study

The current systematic review and meta-analysis aimed to critically evaluate the state of the psilocybin-for-depression RCT literature. Specifically, we aimed to:

1. Systematically assess harm reporting and bias risk within extant psilocybin-for-depression RCTs.
2. Review the degree to which theoretically derived therapeutic action mechanisms are discussed within the psilocybin-for-depression RCT literature.
3. Ascertain incremental efficacy of psilocybin relative to control conditions by comparing pooled effect sizes of depression reduction across RCT conditions.

## Methods

### Search strategy

**Fig. 1:**
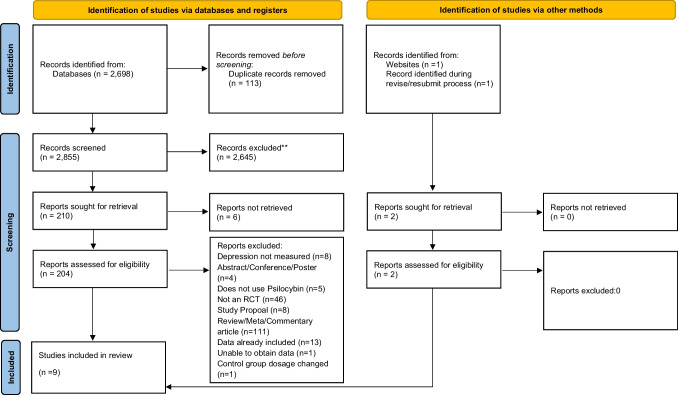
PRISMA 2020 flow diagram for new systematic reviews which included searches of databases, registers and other sources

The Preferred Reporting Items for Systematic Reviews and Meta-Analyses (PRISMA; Page et al. 2021) guidelines were followed for this study (see Fig. 1 for study selection flowchart). The databases used to gather the articles were PsycINFO, CINAHL, Embase, Medline, Web of Science, and Scopus. Search terms “Psilocybin” or “Psychedelic” were paired with “Depression”, and "Randomized Controlled Trial" or “RCT”. Search terms were identical per search engine. The search included all published research through March 2024^a^.

### Inclusion/Exclusion Criteria

The following inclusion criteria were utilized: a) human subjects research, b) psilocybin administration, c) inclusion of participants in a control condition, d) inclusion of at least one validated measure of depression, e) RCT design component (cross-over designs were included for data gathered prior to the cross over), and f) sufficient information available to extract effect sizes (pre/post between-subjects *n*’s, means, and standard deviations).

Exclusion criteria were a) secondary analyses of original RCT data, b) non-peer review publication outlet, and c) psilocybin only control conditions (i.e., dosage studies). English language publication was *not* an exclusion criterion.

### Data extraction and study selection criteria

After completing the initial literature search, titles and abstracts were assessed. If the abstract suggested the study would meet inclusion criteria, the full text was reviewed by a team member. Studies that met inclusion criteria had means (mean change when raw means were not available), standard deviations/errors, *n*’s, therapeutic and pharmacological mechanisms, and basic demographic information extracted for analyses. Any extraction/coding issues were resolved via group discussion. When data were unclear or unavailable, corresponding authors were emailed. Consistent with Yu et al. (2022), if we could not obtain specific estimates, we extracted estimates from figures using the Web Plot Digitizer tool (version 4) to estimate means and deviation estimates (Automeris LLC. 2024). We also tested the Web Plot Digitizer tool on our charts and observed the tool to be sufficiently accurate (largest error observed was within 0.02). To minimize researcher bias, the first author did not provide input on coding/extraction decisions besides determining initial criteria.

### Harm reporting

We followed previous review work (Taillefer de Laportalière et al. 2023) by using the 21-item adverse reporting checklist (Ioannidis et al. 2004; Taillefer de Laportalière et al. 2023) informed by the Consolidated Standards of Reporting Trials (CONSORT) Extension of Harms checklist (Ioannidis et al. 2004). Questions were evaluated on a binary scale where “1” indicates they met criteria. Coding was conducted by two trained research assistants, then replicated by two co-authors (TO and JV), then re-evaluated by an additional team members selected from the broader authorship list. Discrepancies were resolved through team discussion. Specific in-text evidence for a score of “1” for each criterion was extracted to aid in replication. Additionally, excerpts were extracted for evidence of partial criteria (these were still scored as “0” if full criteria were not met). Only original manuscript materials were considered during harm reporting review (i.e., supplemental materials were excluded). Scores for each item on the checklist were summed and described as high quality (17–21); moderate quality (12–16); low quality (7–11); or very low quality (0-6; Taillefer de Laportalière et al. 2023).

### Risk of bias

Risk of bias was assessed via the Cochrane’s Risk of Bias 2.0 (Higgins et al. 2019; Sterne et al. 2019). RoB 2.0 is the recommended tool for evaluating likelihood of bias in RCTs. It consists of five risk domains (risk from the randomization process, deviations from the intended intervention, missing outcome data, measurement of the outcome, and reported results), each domain has multiple questions that are measured on a four-level metric from yes to no with a “no information provided” option. The risk of bias for each domain is qualitatively described as low, medium, and high with an overall risk of bias for the study’s effect. The risk of bias by domain and by the study are determined by an algorithm (Sterne et al. 2019) with question-level metrics informing domain metrics, which in turn inform study metrics. However, despite the algorithm being provided by the tool creators, it is appropriate to override the estimate of bias in instances where a particular risk of bias does not appear to be of concern. Or, conversely, when an estimate of a particular risk of bias is concerning, but not detected by the algorithm. Two coauthors (JV and DP) independently conducted risk of bias assessment using the RoB 2.0 tool. Initial interrater reliability for RoB coding was Cohen’s *κ* = 0.57 (*moderate* agreement). Rating of “probable yes” and “probable no” were converged with their respective “yes” and “no” classifications. After discussion and re- review, 100% agreement was achieved.

### Action mechanisms synthesis

We completed a review of all posited therapeutic MoAs reported in the reviewed RCTs. One co-author (DP) reviewed the introduction and discussion sections of each manuscript, extracted the original author wordings that were used to describe how psilocybin reduced depression. Separate classifications/appraisals were afforded for neurophysiological (e.g., neurotransmitter mechanisms) and psychological mechanisms (e.g., spirituality mechanisms). Co-authors (TO and JV) then independently reviewed and coded each RCT as either "Highly Specific", "Moderately Specific", “Vague”, or "NA" based on each manuscript's level of detail of the extracted MoA(s). The highest codes received by a study’s MoA description became the study’s overall classification for that type of mechanism. Discrepancies between the reviewers were settled by team discussion between DP, JV, and TO.

### Incremental efficacy data analysis

Effect sizes were analyzed using Comprehensive Meta-Analysis (v4) software (Borenstein et al. 2013). All analyses were modeled under random effects. Hedges’ *g* was selected as the index of effect size to adjust for the relatively low *n*’s observed across many of the study conditions. Post-score standard deviation was used to standardize the effect sizes, this was chosen instead of pre-post correlations due to a lack of reporting of pre-post correlations. In all analyses, we examined the *incremental* effect sizes of psilocybin intervention relative to control. That is, we compared the within group change across timepoints between the groups. Incremental Hedges’ *g* represents the strength of the treatment group (psilocybin) relative the control in reducing depression in standard deviation units. Consistent with (Pizer et al. 2024), we adopted Ferguson’s (2009) recommendation of Hedges’ *g* ≥ 0.41 as the threshold for minimal “practical” significance.

Indicators were coded such that positive values represented a greater therapeutic effect in the psilocybin conditions relative to controls (greater depression *reduction*). We conducted meta-analyses where all psilocybin conditions were compared to a novel control condition with time points and depression measures nested within study subgroups. Study subgroups were set as the unit of analysis. Publication bias was statistically assessed using Egger’s regression test, which regresses the effect sizes on the inverse of the standard error. Duval and Tweedie’s trim-and-fill method was used to assess the possibility of missing studies due to publication bias (Duval & Tweedie 2000). Tau (τ) estimates were also calculated to provide heterogeneity context.

### Data availability

 CMA data sheets are available on the Open Science Framework https://osf.io/p3fcx/.

## Results

Study characteristics, including demographic data, are available in Table 1. Overall, *k* = 9 studies met inclusion criteria (see Fig. 1, also see Supplementary File 1 for exclusions). Because one study involved a “high” and a “moderate” psilocybin dose condition, *k* = 10 treatment conditions were evaluated against controls in meta- analyses. In total, a sample of *n* = 602 participants (*n* = 337, 56% in psilocybin conditions) were evaluated.

**Table 1:** Study characteristics

Reference	*n*		Age (Mean)	% Male	%White	Outcome Period**	Funding?	Sample	Financial COI	Psychotherapy Component
	Psilocybin	Control								
Back et al. (2024)	15	15	38	50	25	28 days	Yes	Moderate-to-severe depression symptoms	Yes	Clinical Facilitation
Carhart-Harris et al. (2021)	30	29	43.3	63	93	6 weeks	Yes	Moderate-to-severe MDD	Yes	Clinical facilitation
Davis et al. (2021)	13	11	39.8	33	92	4 weeks	Yes	MDD	Yes	Clinical facilitation
Goodwin et al. (2022)	154*	79	39.8	48	92	3 weeks	Yes	Treatment resistant MDD	Yes	Clinical facilitation
Marschall et al. (2022)	18	23	30.05^b^	38.95^**b**^	NS	3 weeks	No	Attendees of micro-dosing workshops	No	NS
Raison et al. (2023)	51	53	41.1	50	91	43 days	Yes	Moderate-to-severe MDD	Yes	Clinical facilitation
Rosenblat et al. (2024)^**a**^	16	14	44.4	61.3	NS	2 weeks	Yes	Treatment resistant MDD (or BDII)	Yes	Yes
Ross et al. (2016)	14	14^**c**^	56.28	38	90	7 weeks	Yes	Individuals with cancer suffering from AD or GAD	No	Yes
von Rotz et al. (2023)	26	26	36.75	36.55	94.2	2 weeks	Yes	MDD	Yes	Clinical facilitation

*NS* Not Specified, *COI* Conflict of Interest, *MDD* Major Depressive Disorder, *BDII* Bipolar Disorder II, *ASD* Acute Stress Disorder, *AD* Adjustment Disorder, *GAD* Generalized Anxiety Disorder. When overall sample demographics were not reported, the average between groups estimate was calculated (applies to the age, % male, and % White columns). *The 154 represents two groups (10 mg and 25 mg of psilocybin). ^a^ Demographics were collected after assignment, with 31 total participants, however, we include the sample sizes that completed the primary endpoint (30 participants). One participant dropped out prior to treatment, an additional participant dropped out due to adverse events, however it was not specified from which group (29 total participants completed the experiment to the primary endpoint). ^b^ These values were calculated using the full sample from the initial assignment and were retrieved off the OSF repository. ^c^ “They report an additional participant withdrew from the Niacin control group prior to the six-week post first dose assessment in their CONSORT diagram, however in their NCT they list the control group as 15 participants.” **Note also that many authors report gathering additional follow-up information beyond the specified outcome period

Psilocybin dosage modestly varied across conditions with Back et al., (2024), Carhart-Harris et al. (2021), Goodwin et al. (2022), Rosenblat et al. (2024), and Raison et al. (2023) administering 25 mgs of psilocybin. Control conditions were more variable in content and dosage. Three studies used Niacin (i.e. vitamin B3 [differing doses]) as a control (Back et al. 2024; Raison et al. 2023; Ross et al. 2016); one study utilized Mannitol (von Rotz et al. 2023), one study utilized non-psychedelic mushrooms (Marschall et al. 2022); two studies involved waitlist controls (Davis et al. 2021; Rosenblat et al. 2024); one study used an inert psilocybin control (1 mg psilocybin; Goodwin et al. 2022), and one study utilized escitalopram, but also included an inert psilocybin dose (1 mg) for control (Carhart-Harris et al. 2016). Seven of the studies had *n*’s ≤ 30 per condition. Extracted depression measures included: Beck Depression Inventory Amended (BDI-1A; Beck and Steer 1993), 17-item Hamilton Depression Rating Scale (Bech 2010), Montgomery-Åsberg Depression Rating Scale (Montgomery and Åsberg 1979), Quick Inventory of Depressive Symptomatology-Self-Report (Rush et al. 2003), Beck Depression Inventory-II (BDI-II; Beck et al. 1996), Grid Hamilton Rating Scale for Depression (Kalali et al. 2002), Depression Anxiety Stress Scale-21 (Lovibond and Lovibond 1995), Oxford Depression Questionnaire (Price et al. 2012), Hospital Anxiety and Depression Scale-Depression (Zigmond and Snaith 1983), and Symptom Checklist-90-Revised (Derogatis 1977). Time point assessments varied across studies. No study assessed between group outcomes beyond three months. Almost all studies (*k* = 7) reported a financial conflict of interest.

### Harm reporting

Table 2 breaks down harm reporting criteria by each RCT/criterion. Quality was heterogenous. Over half (*k* = 6) the studies were classified as low or very low, one was classified as moderate, and two as high. Criterion 4d was universally missed (i.e., *Described the plan for monitoring for harms and rules for stopping the trial because of harms*). Almost all studies missed criteria 2 (“Information on AEs mentioned in the introduction”), 3a (“Definitions of AEs mentioned”), 3b (“If article mentioned all or selected sample of AE”), 3c (“If article mentioned the use of a validated instrument to report AEs severity”), 4c (Description of how AE were attributed to trial drugs”), 5b (“Description of approach for the handling of recurrent AEs”), 7a (“Provided denominators for AEs”), 8a (“Reported results separately for each treatment arm”), 8c (“Provided both number of AEs and number of patients with AEs”), and 9 (“Described subgroup analysis and exploratory analysis for harms”). Criteria 4b (“Stated the timing of collection of AE data”) and 6b (“Reported deaths and serious AEs”) were almost always met. Marschall et al. (2022) had the most missed scores by a substantial margin. Goodwin et al. (2022) and Raison et al., (2023) had the best harm quality reporting. For a list of specific appraisal criteria see Supplementary File 2. Extracted excerpts from each reviewed manuscript are also provided in Supplementary Files 3–11. We also provide excerpts of when criteria were partially fulfilled, such as in the case of meeting one barrel of a double-barreled question (e.g., criteria 8b “Severity *and* grading of AEs”), and of when authors provided indirect harm reporting evidence (which was still considered insufficient for a favorable score, but could conceivably be linked to harm reporting).

**Table 2:** Harm reporting criteria on the extension of harm checklist

Study	1	2	3a	3b	3c	4a	4b	4c	4d	5a	5b	6a	6b	7a	7b	8a	8b	8c	9	10a	10b	*k*	%	Quality
Back et al. 2024	0	0	0	0	0	0	1	0	0	0	0	1	1	1	1	1	1	0	0	0	0	7	33%	Low
Carhart-Harris et al. 2021	1	0	0	1	0	1	1	0	0	1	0	1	1	1	1	1	0	0	0	1	1	12	57%	Moderate
Davis et al. 2021	0	0	0	0	0	1	1	0	0	0	0	0	1	0	0	0	1	0	0	1	1	6	29%	Very Low
Goodwin et al. 2022	1	0	1	1	1	1	1	1	0	1	0	1	1	1	1	1	1	1	1	1	1	18	86%	High
Marschall et al. 2022	0	0	0	0	0	0	0	0	0	0	0	0	0	0	0	0	0	0	0	0	0	0	0%	Very Low
Raison et al. 2023	1	1	0	1	1	0	1	1	0	1	0	1	1	1	1	1	1	1	1	1	0	17	81%	High
Rosenblat et al. 2024	1	0	0	1	0	1	1	1	0	1	1	0	1	0	1	0	0	0	0	1	1	11	52%	Low
Ross et al. 2016	0	0	0	0	0	0	1	1	0	1	0	1	1	0	0	0	1	0	0	1	1	11	38%	Low
von Rotz et al. 2023	1	0	0	0	0	1	1	0	0	0	0	1	1	0	0	0	0	0	0	1	0	6	29%	Very Low
Total	56%	11%	11%	44%	22%	56%	89%	44%	0%	56%	11%	67%	89%	44%	56%	44%	56%	22%	22%	78%	56%			

See Supplemental File 2 for a full breakdown of specific criteria. See Supplemental Files 3–11 for extracted language from each study

### Risk of bias

Outcomes from the risk of bias review are available in Table 3. Full results including specific RCT-by-criteria rating are available in the Supplementary File 12. RoB 2.0 algorithm results suggested “High” overall bias across all RCTs. Reviewer subjective appraisal concurred with the algorithm that overall risk of bias was high for six studies, though diverged from the algorithm for Back et al. (2024), Goodwin et al. (2022), and Marschall et al. (2022) which we thought would be better classified as having “Some Concerns”. Reasons for bias scores were largely clustered around the *measurement of the outcome* domain. Otherwise, risk of bias was relatively heterogenous across domains/RCTs. Rosenblat et al. (2024) was determined to have the highest risk, being classified as “High” by both the algorithm and assessors across three different domains. Whereas Goodwin et al. (2022) generally evidenced the lowest risk of bias.

**Table 3:** Risk of bias

	Randomization Process		Deviations from Intended Interventions		Missing Outcome Data		Measurement of the Outcome		Selection of the Reported Results		Overall Bias	
	Algorithm Result	Assessor Judgement	Algorithm Result	Assessor Judgement	Algorithm Result	Assessor Judgement	Algorithm Result	Assessor Judgement	Algorithm Result	Assessor Judgement	Algorithm Result	Assessor’s Judgement
Back et al. 2024	Low	Low	Low	Low	Low	Low	High	Some concerns	Low	Low	High	Some concerns
Carhart-Harris et al. 2021	Some concerns	Some concerns	Low	Low	Low	Low	High	High	Low	Low	High	High
Davis et al. 2021	Low	Low	High	High	High	High	High	High	Low	Low	High	High
Goodwin et al. 2022	Low	Low	Low	Low	Low	Low	High	High	Low	Low	High	Some concerns
Marschall et al. 2022	Low	Some concerns	High	High	High	Some concerns	Some concerns	Some concerns	Low	Low	High	Some concerns
Raison et al. 2023	Low	Low	High	High	Low	Low	High	High	Low	Low	High	High
Ross et al. 2016	Some concerns	Some concerns	High	High	Some concerns	Some concerns	High	High	Some concerns	Low	High	High
von Rotz et al. 2023	Low	Low	Low	Some concerns	Low	Low	High	High	High	Low	High	High
Rosenblat et al. 2024	High	High	Some concerns	Some concerns	Low	Low	High	High	High	High	High	High

See supplemental file 12 for additional details

### Action mechanisms

Table 4 illustrates results from the MoA synthesis. Overall, most RCTs provided at least some level of description for neurophysiological and/or psychological MoAs. Ross et al. (2016) and Marschall et al. (2022) were the only studies classified as “Highly Specific” in both domains. Three studies did not specify any neurophysiological mechanisms (Goodwin et al. 2022; Raison et al. 2023; Rosenblat et al. 2024), while Raison et al. (2023) was the only study not to specify a psychological mechanism. For a comprehensive list of extracted MoA statements with accompanying classification, see Supplementary File 13. The most commonly mentioned MoA was action on 5- HT_2A_, though detail regarding how 5-HT_2A_ functioned in relation to depression reduction was variable. Additionally, MoA statements tended to occur in discussion sections (*k* = 24 extracted MoA statements), relative to introductions (*k* = 15; *k* = 6 of which came from Marschall et al. 2022).

**Table 4:** Summary of descriptions of mechanisms of action for psilocybin therapy

Reference	Neurophysiological Mechanism	Psychological Mechanism
Back et al. (2024)	Slightly Specific	Slightly Specific
Carhart-Harris et al. (2021)	Highly Specific	Vague
Davis et al. (2021)	Vague	Slightly Specific
Goodwin et al. (2022)	NA	Vague
Marschall et al. (2022)	Highly Specific	Highly Specific
Raison et al. (2023)	NA	NA
Rosenblat et al. (2024)	NA	Vague
Ross et al. (2016)	Highly Specific	Highly Specific
von Rotz et al. (2023)	Slightly Specific	Slightly Specific

*NA* Not Mentioned. See supplementary file 13 for extracted language that we used to qualify the “Highly Specific, Slightly Specific, and Vague” labels

### Incremental efficacy meta-analyses

**Fig. 2:**
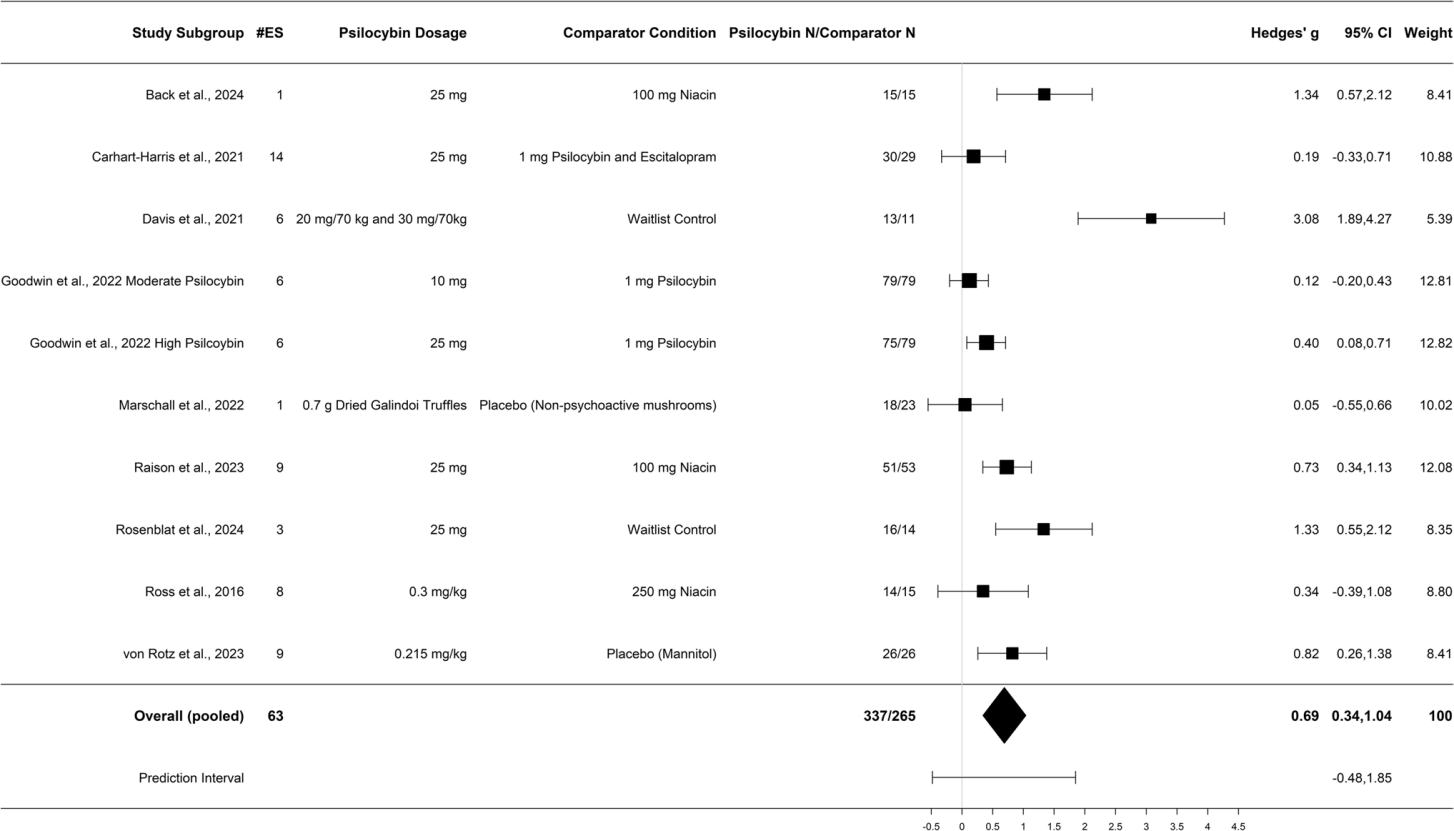
Forest Plot by Subgroup. Note: Goodwin et al. (2022) comparator group is not counted twice in the sample size overall

Random effects meta-analysis of all available effect sizes of psilocybin intervention relative to control conditions suggested a significant incremental effect favoring psilocybin as being statistically superior at reducing depression symptoms, *g* = 0.69; 95% CI = 0.34, 1.04. The results were heterogenous τ = 0.47, *Q*(9) = 39.25, *p* < 0.001, see Fig. 2 for a forest plot of effect sizes. Given the heterogeneity, we provide omnibus results but recommend evaluating each incremental effect sizes to ascertain a comprehensive reflection of the research body. Table 5 provides the incremental effects by study subgroup with relative weights. Supplementary File 14 provides a breakdown of each incremental effect sizes across all RCTs (note that the individual incremental effects in Supplementary File 14 are not adjusted by study-level dependence). Omnibus publication bias estimates indicated a significant correlation between Hedges’ *g* and standard error (Egger’s *b*0 = 3.64, *SE* = 1.36, *t*[8] = 2.68, *p* = 0.014), such that smaller studies tended to yield larger effects that favored psilocybin. The trim-and-fill procedure removed one study, giving an adjusted incremental g = 0.56 95% CI = 0.16, 0.95. Subgroup-analyses indicated the effect was driven by studies where psilocybin was compared against non-intervention controls: *g* = 0.76 95% CI = 0.37, 1.14, relative to active controls (i.e., escitalopram/inert psilocybin; Carhart-Harris et al. 2021), which demonstrated a non-significant incremental effect: *g* = 0.21; 95% CI = −0.31, 0.73. Because Marschall et al. (2022) examined microdosing, we conducted a sensitivity analysis with their study removed. However, omnibus results did not measurably change *g* = 0.76; 95% CI = 0.39, 1.14.

**Table 5:** Incremental effects by study subgroup

Reference	Psilocybin Dose	Control Type	Measures Used	Time Points	*n*		Hedges *g*	95% CI	Relative Weight
					Psilocybin	Control			
Back et al. (2024)	25 mg	100 mg Niacin	MADRS	28 days	15	15	1.34*	0.57, 2.12	8.41
Carhart-Harris et al. (2021)	25 mg	1 mg Psilocybin 10 mg Escitalopram	BDI-1A HAM-D-17 MADRS QIDS-SR-16	3–42 days	30	29	0.19	−0.33, 0.71	10.88
Davis et al. (2021)	20 mg/kg dose 1 30 mg/kg dose 2	WLC	BDI-II GRID-HAMD QIDS-SR	35–56 days	13	11	3.08*	1.89, 4.27	5.39
Goodwin et al. (2022)	10 mg 25 mg	1 mg Psilocybin	MADRS	21 days	79 75	79	0.12 0.40*	−0.20, 0.43 0.08, 0.71	12.81 12.82
Marschall et al. (2022)	.7 g of psilocybin Galindoi truffles	Placebo (mushrooms)	DASS-21	21 days	18	23	0.05	−0.55, 0.66	10.02
Raison et al. (2023)	25 mg	100 mg Niacin	MADRS ODQ Symptoms	1–43 days	51	53	0.73*	0.34, 1.13	12.08
Rosenblat et al. (2024)	25 mg	WLC	MADRS	14 days	16	14	1.33*	0.55, 2.12	8.35
Ross et al. (2016)^a^	.3 mg/kg	250 mg Niacin	HADS-D BDI	2–14 days	14	15	0.34	−0.39, 1.08	8.80
von Rotz et al. (2023)^a^	.215 mg/kg	1 or 5 mg Mannitol	BDI SCL-90-R MADRS	14 days	26	26	0.82*	0.26, 1.38	8.41
Omnibus Effect	-	-	-	-	337	265	0.69*	0.34, 1.04	100

*Significance at the level of *p* < .05. ^a^Baseline values scores are referenced against are 1-day pre psilocybin administration, both studies included a several week pre-psilocybin administration baseline. *WLC* weight list control, *BDI-1A* Beck Depression Inventory Amended, *HAM-D-17* 17-item Hamilton Depression Rating Scale, *MADRS* = Montgomery-Asberg Depression Rating Scale, *QIDS-SR-16* 16-item Quick Inventory of Depressive Symptomatology—Self-Report, *BDI-II* Beck Depression Inventory II, *GRID-HAM* Grid Hamilton Rating Scale for Depression, *QIDS-SR* Quick Inventory of Depressive Symptomatology—Self-Report, *DASS-21* Depression Anxiety Stress Scale-21, *ODQ* Oxford Depression Questionnaire, *HADS-D* Hospital Anxiety and Depression Scale-Depression, *SCL-90-R* Symptom Checklist-90-Revised

## Discussion

The current systematic review and meta-analysis aimed to critically evaluate the state of the psilocybin-for-depression RCT literature. Our findings provide an overview of between group RCTs designed to test the efficacy of psilocybin as a depression intervention. We specifically provide harm reporting, risk of bias, and MoAs syntheses to help contextualize parametric incremental effect results.

Harm quality reporting was heterogenous across RCTs. Overall, there is room for improvement for how psilocybin researchers conceptualize, measure, and report AEs. There was a general lack of discussion of AEs relative to potential benefits, AE materials were often relegated to supplementary materials (hence lower quality scores on otherwise well conducted trials), and a tendency to only vaguely report how AEs were assessed. Frequently, some AE-related information was provided, but it was often unclear how it fit into the broader study. For instance, a tendency to employ a suicidality assessment or blood pressure monitoring as part of the protocol but not include formal (or at least explicate in publication) how additional potential AEs were being assessed, scored, and/or conceptualized.

Across studies, AE definitions were inconsistent. Indeed, this is an area of heterogeneity across the field as researchers conceptualize AEs differently. For example, von Rotz et al. (2023) explicitly excluded “*transient symptoms directly related to the well-known psychotropic effects of psilocybin*” (see their supplementary materials). Whereas, in Davis et al. (2021) included AEs such as “visual distortion” and “altered body sensations”. Echoing previous researchers (Breeksema et al. 2022), consensus over what is considered an AE in the context of psilocybin research is needed. This could explain the diversity of AE measures employed across trials. At times well established AE tools were employed, but authors failed to specify key elements of the CONSORT tool. For example, Carthart-Harris et al. (2021) employed the Medical Dictionary for Regulatory Activities in their study, but did not specify how AE severity was assessed (e.g., criteria 3c). Additionally, most studies failed to adequately describe plans for monitoring AEs and/or criteria for ending a trial due to AEs. Most studies also failed to report both the number of AEs along with the number of patients who experienced AEs. Ideally, ideographic data could be provided where the frequency and AE descriptions are provided for each participant (at least in supplementary contexts) to accompany full sample/subsample estimates.

Despite areas of concern, most studies did report *some* AE information in their main texts and most attempted to provide a balanced view of harm versus benefits in their discussions. It also somewhat understandable that AE information was relegated to supplementary contexts, as few studies explicated AE assessment as a targeted aim. Together, stakeholders could find AE information if they knew where to look. Additionally, the overly low scores for Marschall et al. (2022) should be interpreted cautiously as their study did not target a clinical population, despite assessing depression and utilizing a placebo-controlled RCT design. They were also the only study to implement psychedelic mushrooms. Qualitatively, they were quite different from the other trials being more directed toward basic science than a generalizable intervention.

Risk of bias was also a problem across the reviewed studies. Notably, our risk of bias findings converged with Hovmand et al. (2023), who also analyzed risk of bias for Ross et al. (2016), Carhart-Harris et al. (2021), and Goodwin et al. (2022). Both our team and Hovmand et al. (2023) classified Ross et al. (2016) and Carhart-Harris et al. (2021) as “High” overall risk of bias. They rated Goodwin et al. (2022) as “Low Risk,” whereas we rated them as “Some Concerns” (i.e., assessor’s judgement; algorithm result was high). Our absolute rating of Goodwin et al. (2022) diverged based on the “Measurement of the Outcome” domain. Participant de-blinding (due to psychedelic drug effects or lack thereof) could have influenced the assessment of the outcome despite Goodwin et al.’s (2022) deployment of rigorous outcome-assessment procedures (e.g., initial blinding, naïve raters).

Our outcome measurement classifications (which we almost universally categorized as a high bias source) were anchored in blinding concerns. This is a broader problem with psychedelic research. Although clinician-rated instruments such as the MADRS were employed, the information that the clinician has available to score are driven by participants’ reports of inner experiences (e.g., inner tension, concentration difficulties) or behaviors that cannot be observed in the clinic or over the phone (e.g., reduced sleep; see Montgomery & Åsberg 1979). Therefore, bias in the “Measurement of the Outcome” domain is susceptible to the compound influence of the patient’s self-report and the clinician’s interpretation. Similar to responding in a self-report format, responding during the MADRS (and other clinician-scored instruments that rely on participant self-report) are susceptible to de-blinding by the subjective effects of psilocybin. That is, even if the assessor was blind, the patient could be de-blinded by virtue of the psychedelic experience, in turn de-blinding the assessor. Therefore, for our RoB analysis, we diverged from considering the “assessor” as the “observer” for the rater-administered MADRS. We consider the participant to be the observer and encourage other evaluations of psychedelic substance clinical trials that rely on patient self-reported information to do the same.

Ideally, RoB analyses should be expanded to model the compound sources of bias. Participant de-blinding issues, which pervade psychedelic science (see Muthukumaraswamy et al. 2021; Nayak et al. 2023), imply that evidence produced from psychedelic RCTs using extant control methods (e.g., inert placebos, simple active placebos) may be no more rigorous than evidence produced from non-RCT trials (i.e., open-label studies). That is, a therapeutic effect is evident, but it is difficult to ascertain how much of that effect is due to de- blinding/expectancy/placebo excitement over a novel therapy. Extant RoB measures must be considered with such limitations in mind.

Conversely, we rated Marschall et al. (2022) as only having “Some Concerns” for the “Measurement of the Outcome” domain. In contrast to the problems with high-dose psychedelic trials outlined above, the reduced risk of bias was primarily driven by their aim of investigating microdosing. Because a paucity of perceived subjective effects is part of the definition of microdosing (Kuypers et al. 2019), it is easier to effectively blind microdose trials. Importantly, we are not suggesting that microdosing, as currently studied and practiced is completely subperceptual (Fadiman & Korb 2019; Holze et al. 2021). However, we suggest it is easier to create non-psychedelic blinding conditions that mimic “improved energy” (Anderson et al. 2019) vs. “I approached the border where existence began, and on the other side of this border was nothing” (Noorani et al. 2018). We encourage investigators to further consider trials testing microdosing, as this may confer an easier scientific problem to initially solve. Should microdosing trials maintain blindness and find efficacy, it will be clearer that the treatment effects are not driven by de-blinding.

Researchers varied in the degree to which they specified/considered MoAs. Additionally, the nature of the reported MoAs differed by research team. This likely reflects the various MoAs posited in the psilocybin literature. Psilocybin’s agonist activity on serotonergic receptors (particularly 5-HT_2A_) was arguably the most commonly posited MoA (with varying degrees of specificity; see Supplemental File 13). Other mechanisms included the potential for increased interoceptive awareness, neural plasticity, and network level changes, among others. Most researchers did not integrate multiple MoA theories, such as how 5-HT_2A_ activity might yield changes in the default mode network (Gattuso et al. 2023; Smigielski et al. 2019) though exceptions were evident (Ross et al. 2016, see Supplementary File 13). Few of the research teams measured any a priori MoAs or conducted any meaningful mediation analyses within the reviewed RCTs.

One potential reason for the lack of MoA tests was that such investigations occurred in post hoc publications. We attempted to confirm all such cases. Of those, Daws et al. (2022) stands out as an example of how researchers are trying to answer MoA questions. They compared brain states using fMRI in participants from Carhart-Harris et al. (2021). Their results provided evidence that participant response to psilocybin was correlated with increased brain “network flexibility” relative to an absence of commensurate brain state changes in participants in the escitalopram condition. However, their relatively small sample sizes were each further reduced by attrition (e.g., “head motion”), meaning effect sizes were probably inflated and replication probability reduced (Marek et al. 2022). Other follow-up studies from the Carhart-Harris et al. (2021) sample include Zeifman et al (2023) who conducted a study suggesting reductions in experiential avoidance as a potential mechanism. Murphy et al. (2022) also conducted a follow-up, Carhart-Harris (2016) and highlighted the importance of therapist rapport/therapeutic alliance in psilocybin intervention. Their results are consistent with a common factors model (Wampold 2015) that if alliance is sound, therapeutic effects should follow.

Several other secondary analyses were also conducted on the reviewed RCTs. Broadly, these did not include traditional mediation-based MoA analyses. For example, Goodwin et al. (2023) published a follow-up analysis based on the Goodwin et al. (2022) sample, though this did not involve MoA/mediation analyses. Goodwin et al. (2025) also recently published a follow-up analysis suggesting dosage strength is correlated with psychedelic psychological experiences (e.g., “Oceanic Boundlessness”) which could potentially explain reductions in depression. Malone et al. (2018) published a follow-up to Ross et al. (2016); however, no formal MoA analyses were conducted. Though they did reference the MoAs discussed in the original Ross et al. (2016) study. Gukasyan et al. (2022) conducted a follow-up on Davis et al. (2021) but again there was no formal MoA analyses, but rather bivariate correlations with various potential mechanisms such as spiritual significance and mystical experience (which could be conceptualized as “outcomes” instead of MoAs based on their analyses). Jungwirth et al. (2024) recently published a follow-up to von Rotz et al. (2023) with analyses suggesting psilocybin may increase empathy, thereby indirectly reducing depression. We were unable to identify any potential MoA secondary analyses that were specific to psilocybin’s effect on depression for the other reviewed RCTs (Back et al. 2024; Marschall et al. 2022; Raison et al. 2023; Rosenblat et al. 2024).

The general lack of MoA measurement signifies the neophyte nature of psilocybin intervention. We encourage authors to proactively specify MoAs and report MoA assessment in primary publications. The potential concern when MoA analyses are absent is that psilocybin is presented as a treatment without a clear argument for why it should reduce depression. This is consistent with our observation that researchers tended to discuss MoAs in their discussion sections rather than their introductions (i.e., backwards theorizing). Indeed, many researchers relied on citations of prior studies that indicate psilocybin can be associated with some degree of mood enhancement rather than trying to identify an explanatory process (see Supplementary File 13). By demonstrating the mediational mechanisms involved in psilocybin intervention, arguments for placebo and expectancy effects related to psilocybin as a novel (if not exciting) intervention become less profound. Another concern is that it does not appear that researchers performing secondary analyses in subsequent publications conducted appropriate Bonferroni corrections to account for the number of analyses already performed. This problem is exacerbated by the fact that all of the RCTs had small samples. Said another way, there is significant type 1 error risk. While an understandable starting place, more empirically based testing of MoAs is needed in original trials to demonstrate psilocybin is uniquely responsible for the reported depression reductions and not expectancy.

This could be done with a variety of approaches depending on the MoAs of interest. For example, if 5- HT_2A_ activation is theorized to be causing the therapeutic effects, researchers should have evidence of baseline 5-HT_2A_ problems (however defined) and post-test evidence of sustained 5-HT_2A_ change corresponding to reduced depression scores. Alternatively, similar to the approach taken by Daws et al. (2022), participants could undergo imaging procedures before and after the intervention to see how neural changes correspond to depression changes. Further controls could be added, such as non-pathological participants. That is, would brain networks resemble healthy controls before/after treatment? Future researchers should also consider assigning patients to treatment groups based on MoA evidence. It is possible that psilocybin could have meaningful therapeutic effects for some, but not all, depression etiologies. If group assignment is based solely on latent disease classification (e.g., meeting DSM-5- TR criteria), then there is no way of knowing which patient might respond (positively or adversely) as the syndromal classification confounds etiology. Psilocybin researchers could categorize depressive participants with more specificity (ideally, by operationalized MoAs) to correct for such confounding issues. This recommendation is consistent with recent paradigm shifts within clinical science, such as the Research Domain Criteria (Insel et al. 2010).

To be sure, MoA limitations is an underlying problem in psychiatry research and is not necessarily specific to psilocybin intervention (Cuijpers & Cristea 2015). We also recognize that understanding the exact MoAs might not be essential if the therapeutic effects are robustly efficacious. Our results suggest some degree of incremental efficacy exists, but it is relatively modest and needs to be contextualized by the methodological limitations (i.e., small sample sizes biasing effect sizes), generalizability issues, and researcher bias (prominence of financial conflicts of interest). Thus, a robust explanation for why psilocybin could specifically reduce depression notwithstanding confounding factors, would greatly enhance stakeholder trust in the intervention.

In terms of our incremental meta-analyses, psilocybin statistically reduced depression relative to controls, but only to a moderate degree. Taking the BDI-II (one of the most popular depression measures assessed across RCTs) as an example, psilocybin on average would reduce depressive symptoms by approximately 7.9 points more than the reduction achieved by controls (the BDI-II has a range from 0 to 63). This estimate is based on the BDI-II outpatient normative data (Beck et al. 1996). Thus, some unique potential is evident. However, almost all the studies used non-psychotropic agents as controls. Carhart-Harris et al. (2021) was the only exception, demonstrating a non-significant incremental effect. Observation of study-by-study effects suggests the strongest effects come from studies like Davis et al. (2021; incremental *g* = 3.08) and Rosenblat et al. (2024; incremental *g* = 1.33), which utilized waitlist controls and had extremely small samples (i.e., unstable metrics/effect size inflation). Conversely, Goodwin et al. (2022), a relatively larger study, demonstrated much more modest incremental effects (*g* = 0.19 for 10 mg psilocybin and *g* = 0.43 for 25 mg psilocybin). The Egger’s test demonstrated a significant correlation between standard error (small sample *n*) and effect size, favoring psilocybin. Taken together, our results indicate unique therapeutic effects are observed but also raise decline effect concerns. In other words, future large scale (*n*’s > 100 per group) psilocybin-for-depression RCTs that utilize active controls (FDA approved antidepressants) will likely be associated with a reduction in incremental effect. The primary question is how much of an effect size reduction will occur? Concurrently, how long will the incremental effect last? No studies followed between group outcomes beyond three months.

That said, psychedelic-assisted therapy is a complete paradigm shift for psychiatry. By design, psychedelic-assisted therapies entail the deemphasis of long-term pharmacological treatments and substitute an emphasis on the healing power of the pharmacologically induced *acute* experience. This shift implies concomitant new ways of thinking about psychiatric diagnosis, etiology, and therapeutic MoAs (Schenberg 2018). In other words, even if psilocybin only yields modest incrementally superior effects relative to standard treatments, it could still be globally superior if intervention was needed significantly less frequently than a daily prescription. If the theorized single session/single dose approach yields long lasting reductions in depressive symptoms, thereby reducing side effects and other problems traditionally associated with typical psychopharmacological agents, psilocybin may hold substantially more incremental value than statistically demonstrated.

### Observations

There were a relatively high number of barriers to conducting the current meta-analysis. First, few of the relevant studies comprehensively provided needed statistical information in original texts. This is problematic as clinicians and researchers need such information when trying to appraise this body of work. Underreporting in original texts is somewhat understandable, as comprehensive results must often be relegated to supplementary files (the current paper serves as a prime example). However, many of the supplementary files reviewed lacked necessary information such as depression outcome means at specific time points and deviation estimates. Many authors reported pieces of information in the form of charts, but not exact estimates, and some failed to provide necessary information (e.g., exclusion of Grob et al. 2011, see Supplementary File 1). In many cases, enough results are provided such that analyses were possible, but hints towards unreported data were evident. For instance, Back et al. (2024), collected between-group measurements at days “1, 8, 15, and 28” (p. 4). However, they only reported means and standard deviations at baseline and day 28. We attempted to contact many authors to obtain such information, but received few responses, with only a subset of authors providing further data. Supplementary File 15 provides a record of our attempts to contact corresponding authors of the evaluated RCTs. Four teams failed to respond to multiple inquiries, and two responded after multiple prompts. Our efforts to contact authors included attempts to clarify important pieces of information. For example, we observed in the pre-registration for von Rotz et al. (2023; ClinicalTrials.gov NCT03715127) that they initially planned to report depression outcomes at Day 32, yet their publication only presents results up until day 14. There is no documented explanation for this discrepancy. We recommend all psilocybin researchers report means, deviation estimates, and *n*’s for participants in each treatment and control group for all time points in their studies, in addition to other useful statistical information (95% confidence intervals for respective point estimates, *p*-values).

Almost all of the RCTs had authors who reported financial conflicts of interest with companies sponsoring psilocybin intervention. We are concerned that such financial conflicts of interest may be adding an additional source of bias. Indeed, this is a well-established corollary of misleading findings that favor respective target pharmacologic agents (Bekelman et al. 2003; Ioannidis 2005; Mahase 2024; Nejstgaard et al. 2020). It would be preferable for analyses to be conducted and reported by researchers who are not funded by private companies interested in psychedelic therapies.

### Limitations

Our study presents a critical evaluation of the psilocybin-for-depression research program. We offer this synthesis of the research in the spirit of improving psychedelic research. Our study is limited by the relatively small literature body. We observed many interesting trends that could be important moderators. For instance, the microdosing study by Marschall and colleagues (2022) failed to yield a significant incremental effect, but the absence of additional microdosing RCTs prevented us from conducting meaningful meta-regression analyses to evaluate this finding. We also observed that most of the RCTs predominantly included White participants. Presently, psychedelic science is grappling with multicultural, diversity, and social justice issues. Indeed, in contemporary psychedelic science, participants have been relatively demographically homogeneous (Michaels et al. 2018). This limits knowledge, makes the external validity of research findings uncertain (Ortiz et al. 2022), and stunts our understanding of the ethnopsychopharmacology of psychedelics and psychedelic-assisted therapies (Fogg et al. 2021). These inequities are exacerbated by the well-documented need for novel treatments in diverse communities, the rich history of psychedelic use in non-White, indigenous cultures, and the unethical practices that harmed vulnerable groups during prior waves of psychedelic research (Strauss et al. 2022). Future researchers should make greater efforts to include diverse participants in their RCTs.

We were unable to disentangle the role of psychotherapeutic facilitation in the intervention. This was one of many issues that recently precluded favorable ratings from the FDA for MDMA PTSD interventions. Some degree of facilitation was apparent in almost every reviewed study, with multiple studies acknowledging concurrent psychotherapy. Ostensibly, this issue should be controlled by the RCT methodologies. However, that is assuming all participants received equal degrees of psychotherapy quality (in terms of alliance, trust, experience, etc.) in each condition per study. As such, it is difficult to ascertain what effects are attributable to psychotherapy and which are attributable to psilocybin, as different therapists might have different skills and levels of patient alliance (that in turn differs by patient). This issue is exacerbated by the noted blinding problem, with therapists being de-blinded by participant reactions, thereby altering their therapeutic approaches between conditions. As noted by Murphy et al. (2022), therapist alliance plays a significant role in psilocybin’s therapeutic effectiveness.

## Conclusions

Psilocybin intervention demonstrates a significant incremental therapeutic effect that is modestly superior to control conditions in treating depression. Decline effects linked to expectancy/placebo effects are a concern within the psilocybin-for-depression research program. Better controls and larger samples sizes could attenuate effect sizes in future RCTs. Harm reporting is highly variable and largely driven by how researchers define and report AEs, as well as their strategies for managing AEs. Risk of bias can be reduced by employing more objective outcome measurements that are not reliant on patient self-report. MoAs were highly variable, and more often specified in discussions opposed to introductions, and generally not measured in primary or even secondary analyses. We advise psilocybin researchers to specify mechanisms, *a priori,* that theoretically explain therapeutic effects in depression treatment. Similarly, psilocybin researchers are encouraged to measure mechanisms and have inclusion criteria based on hypothesized mechanisms (not necessarily latent disease classification). Psilocybin researchers are also encouraged to conduct biomarker-driven causal mediational analyses (Muthukumaraswamy 2023). This would involve: 1) assessing pre-trial expectancies; 2) capturing known objective correlates of outcomes associated with psychedelics (e.g., biomarkers); and 3) measuring treatment outcomes. These data can be combined to statistically parse the causal effects of psychedelics vs. expectancy (placebo) in a mediation model based on the logic that expectancies are more likely to influence self-report outcomes than observable biomarkers. We also encourage funding bodies to create accessible psychedelic-focused mechanisms for researchers not affiliated with for-profit psychedelic promoting companies. This will allow for more teams to conduct rigorous psychedelic research, while minimizing bias associated with private funding. It is hoped that by applying these recommendations the psilocybin research program can improve, such that it can be utilized in a way that maximizes beneficence and minimizes error.

## Supplementary Information

Below is the link to the electronic supplementary material.

Supplementary file1 (DOCX 19 KB)

Supplementary file2 (DOCX 18 KB)

Supplementary file3 (DOCX 57 KB)

Supplementary file4 (XLSX 19 KB)

Supplementary file5 (DOCX 40 KB)

Supplementary file6 (DOCX 36 KB)

Supplementary file7 (DOCX 16 KB)

## Data Availability

Data is available from the first author upon request.
