# Correction to: Incremental efficacy systematic review and meta-analysis of psilocybin-for-depression RCTs

**DOI:** 10.1007/s00213-025-06818-7

**Published:** 2025-05-17

**Authors:** Nicholas C. Borgogna, Tyler Owen, Dan Petrovitch, Jacob Vaughn, David A. L. Johnson, Louis A. Pagano, Stephen L. Aita, Benjamin D. Hill

**Affiliations:** 1 Department of Psychological Sciences, Texas Tech University, Lubbock, TX USA; 2 Department of Psychology, University of Alabama at Birmingham, Birmingham, AL USA; 3 St. Cloud VA Health Care System, St. Cloud, MN USA; 4 VA Maine Healthcare System, Augusta, ME USA; 5 Department of Psychology, University of Maine, Orono, ME USA; 6 Alabama Department of Psychology, University of South, Mobile, AL USA

**Correction to: Psychopharmacology**

10.1007/s00213-025-06788-w

In the published paper, there were errors with an author’s name and an institutional affiliation. The authorship should have been written as the following: “Nicholas C. Borgogna, Tyler Owen, Dan Petrovitch, Jacob Vaughn, David A. L. Johnson, Louis A. Pagano, Jr., Stephen L. Aita, and Benjamin D. Hill.” The third institutional affiliation should have been written as follows: “St. Cloud VA Health Care System, St. Cloud, MN, USA.

The original article has been corrected.

