# Supplementary material for: Incremental efficacy systematic review and meta-analysis of psilocybin-for-depression RCTs
Source: Psychopharmacology (Berl). 2025 Apr 23;242(10):2139–57. doi: 10.1007/s00213-025-06788-w (PMC12449434; doi:10.1007/s00213-025-06788-w)
Supplement: Supplementary file 1 — Supplementary file1 (DOCX 19 KB) [file 213_2025_6788_MOESM1_ESM.docx]

Supplementary File 1

Exclusions of prominent studies

Exclusion of Griffiths et al. (2016)

Griffiths et al. (2016) was excluded due to dosage modifications in both conditions, but particularly the control group. The original dosage for the experimental group was 30 mg/70kg but was reduced in three participants to 22 mg/70 kg. The reason for the reduction was due to adverse events causing two participants of the first three examined to drop out. The original dose for the control group was 3 mg/70 kg, this was dropped to 1 mg/70 kg after 12 participants experienced potentially experimental level psychedelic effects. The intention of the control group in Griffiths et al., 2016 was to have a small dose of psilocybin that would have no impact on the participant, effectively working to retain the blind throughout the study. This practice has been done in other RCTs of psilocybin’s effect on depression (see Carhart-Harris et al., 2021; Goodwin et al., 2022). However, these contemporary studies used 1 mg at a fixed bodyweight, with the assumption that it is an inert dose. Because of the change in doses in Griffiths et al. (2016), almost half of their sample (46%) received a dose that was close to an experimental level dose. Thus, we opted to exclude this study from analyses.

Exclusion of Grob et al. (2011)

Grob et al. (2011) is one of the most prominent RCTs within the psilocybin for depression literature. They included the BDI as an outcome marker in their study. However, they never provide specific BDI means, variance estimates or *n*’s for each group by time point. We initially extracted estimates from their figure 3a using our digital extraction tool (Automeris LLC., 2024). However, the following wording in their text gave us pause for inclusion:

“*For the BDI, there was an overall interaction of psilocybin and day that approached but did not attain statistical significance (F_1,11_ = 3.75, P = .08). There was no appreciable change from 1 day prior to placebo administration to 2 weeks after experimental treatment, whereas a trend was observed after psilocybin administration, from a mean (SEM) score of 16.1 (3.6) one day before treatment to 10.0 (2.7) two weeks after treatment (*[*Figure 3*](https://jamanetwork.com/journals/jamapsychiatry/fullarticle/210962#yoa05049f3)*A).*”

Particularly, the *F* statistic with the noted degrees of freedom suggest that the estimates in figure 3A were within group changes of all participants (*n*=12) who had received both treatments (psilocybin and the control niacin) rather than psilocybin (*n*=6) compared to niacin (*n*=6) estimates prior to their cross-over. A potential interpretation of figure 3A is the BDI changes by groups (*n*=6 each) over the course of two weeks, which would meet inclusion for our study. However, as noted, the wording in their document suggests the estimates in figure 3a represent aggregate group (*n*=12) changes after participants had each respective intervention. This would disqualify them from our study since we would not have pre-crossover estimates. Because the authors are unclear about their estimates, we opted to exclude them. We ran analyses with Grob et al. (2011) included and our findings do not measurably change (contact first author for specific estimates). We attempted to contact Grob et al. (including many of the co-authors), on this and many other issues, but never received a reply (see Supplementary File 15).
