# Supplementary material for: Incremental efficacy systematic review and meta-analysis of psilocybin-for-depression RCTs
Source: Psychopharmacology (Berl). 2025 Apr 23;242(10):2139–57. doi: 10.1007/s00213-025-06788-w (PMC12449434; doi:10.1007/s00213-025-06788-w)
Supplement: Supplementary file 2 — Supplementary file2 (DOCX 18 KB) [file 213_2025_6788_MOESM2_ESM.docx]

| Supplemental File 3 | | |
| --- | --- | --- |
| Quality of Harms Reporting Criteria (Ioannidis et al., 2004). | | |
| Section of Paper | CONSORT Harm Recommendations | Detailed Items |
| Title and Abstract | If the study collected data on harms and benefits, the title or abstract should so state. | 1. AEs mentioned in title or abstract |
| Introduction | If the trial addresses both harms and benefits, the introduction should so state. | 2. Information on AEs mentioned in the introduction |
| Methods | Include a list of AEs with definitions for each (with attention, when relevant, to grading, expected v. unexpected events, references to standardized and validated definitions, and description of new definitions). | 3a. Definitions of AEs mentioned |
|  |  | 3b. If article mentioned all or selected sample of AE |
|  |  | 3c. If article mentioned the use of a validated instrument to report AEs severity |
|  | Clarify how harms-related information was collected (mode of data collection, timing, attribution methods, intensity of ascertainment, and harms-related monitoring and stopping rules, if pertinent). | 4a. Describe the mode of data collection (e.g. diaries, phone interviews, face-to-face interviews) |
|  |  | 4b. Stated the timing of collection of AE data |
|  |  | 4c. Description of how AE were attributed to trial drugs |
|  |  | 4d. Described the plan for monitoring for harms and rules for stopping the trial because of harms |
|  | Describe plans for presenting and analyzing information on harms (including coding, handling of recurrent events, specification of timing issues, handling of continuous measures, and any statistical analyses). | 5a. Described the methods for presenting and/or analyzing AEs |
|  |  | 5b. Description of approach for the handling of recurrent AEs |
| Results | Describe for each arm the participant withdrawals that are due to harm and the experience with the allocated treatment. | 6a. Reported withdrawals because of AE in each arm |
|  |  | 6b. Reported deaths and serious AEs |
|  | Provide denominators for describing harms. | 7a. Provided denominators for AEs |
|  |  | 7b. Provided definitions used for analysis set (intention to treat, per protocol, safety data available, unclear) |
|  | Present the absolute risk of each adverse event (specifying type, grade, and seriousness per arm), and present appropriate metrics for recurrent events, continuous variables, and scale variables, whenever pertinent. | 8a. Reported results separately for each treatment arm |
|  |  | 8b. Severity and grading of AEs |
|  |  | 8c. Provided both number of AEs and number of patients with AEs |
|  | Describe any subgroup analysis and exploratory analysis for harms. | 9. Described subgroup analysis and exploratory analysis for harms |
| Discussion | Provide a balanced discussion of benefits and harms with emphasis on study limitations, generalizability, and other sources of information non harms. | 10a. Provided a balanced view that puts benefits and harms into perspective |
|  |  | 10b. Included limitations of study with respect to harms (e.g. lack of power, short duration of exposure, inconclusive findings, post hoc analysis, generalizability of AE info as dependent on clinical setting) |

*Note:* AE=Adverse Events, Table reprinted from “Reporting of harms in clinical trials of esketamine in depression: a systematic review”, by T. Taillefer de Laportalialière, A. Jullien, A. Yrondi, P. Cestac, F. Montastruc, 2023, *Psychological Medicine*, 54, P. 4307. Original source was Ioannidis et al. (2004).
