# Supplementary material for: Incremental efficacy systematic review and meta-analysis of psilocybin-for-depression RCTs
Source: Psychopharmacology (Berl). 2025 Apr 23;242(10):2139–57. doi: 10.1007/s00213-025-06788-w (PMC12449434; doi:10.1007/s00213-025-06788-w)
Supplement: Supplementary file 3 — Supplementary file3 (DOCX 57 KB) [file 213_2025_6788_MOESM3_ESM.docx]

| Supplementary File 3 | |
| --- | --- |
| Back et al., 2024 | |
| Criteria | Example Text w/Page Number |
| 1. AEs mentioned in title or abstract | Not Specified |
| 2. Information on AEs mentioned in the introduction | Not Specified |
| 3a. Definitions of AEs mentioned | Not Specified |
| 3b. If article mentioned all or selected sample of AE | “… adverse events; and suicidal ideation and behavior using Columbia Suicide Severity Rating Scale [score range varies]).” (p.4) |
| 3c. If article mentioned the use of a validated instrument to report AEs severity | *“Measures at the medication session were collected on paper (medication experience using Mystical Experience Questionnaire, 30 Questions [MEQ-30; 0-150, with higher scores indicating more intense symptoms]; adverse events; and suicidal ideation and behavior using Columbia Suicide Severity Rating Scale [score range varies]).” (p.4) |
| 4a. Describe the mode of data collection (e.g. diaries, phone interviews, face-to-face interviews) | *“In addition to the 3 planned integration sessions, additional supportive visits were available.” And “Measures at the medication session were collected on paper (medication experience using Mystical Experience Questionnaire, 30 Questions [MEQ-30; 0-150, with higher scores indicating more intense symptoms]; adverse events; and suicidal ideation and behavior using Columbia Suicide Severity Rating Scale [score range varies]).” (p.4) |
| 4b. Stated the timing of collection of AE data | "Participants received check-in text messages in the evening after their medication session. In addition to the 3 planned integration sessions, additional supportive visits were available.” And “Measures at the medication session were collected on paper … adverse events; and suicidal ideation and behavior using Columbia Suicide Severity Rating Scale [score range varies]).” (p.4) |
| 4c. Description of how AE were attributed to trial drugs | Not Specified |
| 4d. Described the plan for monitoring for harms and rules for stopping the trial because of harms | Not Specified |
| 5a. Described the methods for presenting and/or analyzing AEs | Not Specified |
| 5b. Description of approach for the handling of recurrent AEs | Not Specified |
| 6a. Reported withdrawals because of AE in each arm | “All participants completed all intervention sessions.” (p.5) |
| 6b. Reported deaths and serious AEs | “No serious adverse events occurred.” (p. 8) |
| 7a. Provided denominators for AEs | “All participants completed all intervention sessions. Fifteen participants were randomized to psilocybin and 15 were randomized to niacin” (p.5) and “On the day of the psilocybin administration, other adverse events occurred, such as mild nausea (4 [27%]), mild headache (4 [27%]), mild tachycardia (2 [13%]), and hypertension (mild, 6 [40%], moderate, 8 [53%], or severe, 1 [7%], which resolved in <20 minutes without medical treatment). There were no episodes of psychosis or attempts to leave the room without permission. For the niacin sessions, 1 participant experienced a mild headache.” (p.8) |
| 7b. Provided definitions used for analysis set (intention to treat, per protocol, safety data available, unclear | “All participants completed all intervention sessions. Fifteen participants were randomized to psilocybin and 15 were randomized to niacin” (p.5) |
| 8a. Reported results separately for each treatment arm | “On the day of the psilocybin administration, other adverse events occurred, such as mild nausea (4 [27%]), mild headache (4 [27%]), mild tachycardia (2 [13%]), and hypertension (mild, 6 [40%], moderate, 8 [53%], or severe, 1 [7%], which resolved in <20 minutes without medical treatment). There were no episodes of psychosis or attempts to leave the room without permission. For the niacin sessions, 1 participant experienced a mild headache. After day 0, there were no episodes of thought distortion (derealization or depersonalization) or perceptual disturbances.” (p.8) |
| 8b. Severity and grading of AEs | “No serious adverse events occurred. Other adverse events were managed without medical intervention except for 1 instance of nausea in the psilocybin group. On the day of the psilocybin administration, other adverse events occurred, such as mild nausea (4 [27%]), mild headache (4 [27%]), mild tachycardia (2 [13%]), and hypertension (mild, 6 [40%], moderate, 8 [53%], or severe, 1 [7%], which resolved in <20 minutes without medical treatment). There were no episodes of psychosis or attempts to leave the room without permission. For the niacin sessions, 1 participant experienced a mild headache. After day 0, there were no episodes of thought distortion (derealization or depersonalization) or perceptual disturbances.” (p. 8) |
| 8c. Provided both number of AEs and number of patients with AEs ** | “On the day of the psilocybin administration, other adverse events occurred, such as mild nausea (4 [27%]), mild headache (4 [27%]), mild tachycardia (2 [13%]), and hypertension (mild, 6 [40%], moderate, 8 [53%], or severe, 1 [7%], which resolved in <20 minutes without medical treatment). There were no episodes of psychosis or attempts to leave the room without permission. For the niacin sessions, 1 participant experienced a mild headache. After day 0, there were no episodes of thought distortion (derealization or depersonalization) or perceptual disturbances.” (p. 8) |
| 9. Described subgroup analysis and exploratory analysis for harms | Not Conducted |
| 10a. Provided a balanced view that puts benefits and harms into perspective | Not Specified |
| 10b. Included limitations of study with respect to harms (e.g. lack of power, short duration of exposure, inconclusive findings, post hoc analysis, generalizability of AE info as dependent on clinical setting) | Not Specified |
| AE=Adverse Event | |

*Note*: *Indicates that the authors mentioned information that could conceivably have met this criterion, but the information reported was insufficient. It is not included in the final tally. ** This is a double-barreled question; the authors met a portion of the criteria. It is not included in the final tally.

| Supplementary File 4 | |
| --- | --- |
| Carhart-Harris et al., 2021 | |
| Criteria | Example Text w/Page Number |
| 1. AEs mentioned in title or abstract | “The incidence of adverse events was similar in the trial groups.” (p. 1402) |
| 2. Information on AEs mentioned in the introduction | Not Specified |
| 3a. Definitions of AEs mentioned | Not Specified |
| 3b. If article mentioned all or selected sample of AE | “Other secondary outcomes were the changes from base-line to 6 weeks in the scores on the” … “Suicidal Ideation Attributes Scale (SIDAS), as well as the scores at 6 weeks on the Psychotropic-Related Sexual Dysfunction Questionnaire (PRSexDQ), the Laukes Emotional Intensity Scale (LEIS),17 and the Emotional Breakthrough Inventory,18 which assessed acute subjective experiences after each dosing day (Fig. S4 and Table S5). An investigator-constructed patient-rated scale (the Post-Treatment Changes Scale [PTCS]) was used as a safety outcome measure for assessing post-treatment side effects and other phenomena that previous work has associated with psychedelic compounds or selective serotonin-reuptake inhibitors” (p. 1405) and “Adverse events were recorded at every visit and telephone call from dosing-day 1 through week 6. Adverse events were assessed by asking “how have you been since your last visit?” or on the basis of events that were observed at the trial site. Additional details of the criteria used for the reporting of adverse events are provided in the protocol. All adverse events that occurred or worsened between dosing-day 1 and week 6 were recorded and coded with the use of the Medical Dictionary for Regulatory Activities, version 23.0.” (p. 1405) |
| 3c. If article mentioned the use of a validated instrument to report AEs severity | *“All adverse events that occurred or worsened between dosing-day 1 and week 6 were recorded and coded with the use of the Medical Dictionary for Regulatory Activities, version 23.0.” (p. 1405) |
| 4a. Describe the mode of data collection (e.g. diaries, phone interviews, face-to-face interviews) | “At visit 1 (baseline), all the patients underwent functional MRI, completed a battery of cognitive and affective processing tasks (data not yet analyzed), and attended a preparatory therapeutic session. At visit 2, which occurred 1 day after visit 1, the patients in the psilocybin group received 25 mg of psilocybin, and those in the escitalopram group received 1 mg of psilocybin, which was presumed to have negligible activity (dosing-day 1).” (p. 1404). And “The dosing days for each patient were supervised by the two mental health professionals who had been assigned to the patient. Supervision consisted of caring for the physical and psychological well-being of the patient and responding to signs of patient discomfort during and immediately after the administration of a trial medication.” (p.1404). And “The structure of this visit was similar to that of visit 1 and involved the performance of functional MRI (6 weeks after the first), cognitive and affective processing tasks, final clinician rated assessments, and psychological debriefing.” (p.1405). |
| 4b. Stated the timing of collection of AE data | “Adverse events were recorded at every visit and telephone call from dosing-day 1 through week 6.” (p.1405). |
| 4c. Description of how AE were attributed to trial drugs | Not Specified |
| 4d. Described the plan for monitoring for harms and rules for stopping the trial because of harms ** | “All adverse events that occurred or worsened between dosing-day 1 and week 6 were recorded and coded with the use of the Medical Dictionary for Regulatory Activities, version 23.0.” (p. 1405) |
| 5a. Described the methods for presenting and/or analyzing AEs | “Logistic regression, with adjustment for baseline scores, was used to analyze the secondary outcomes of response and remission according to the QIDS-SR-16, as well as the additional outcomes of response and remission according to the BDI-1A, the HAM-D-17, and the MADRS. The changes from baseline to week 6 in the scores on the HAM-D-17, the QIDS-SR-14, the MADRS, the WEMWBS, the FS, the BEAQ, the WSAS, the SHAPS, the STAI, and the LEIS were analyzed with the use of ANCOVA or repeated-measures ANCOVA, with adjustment for baseline (if possible). The changes from baseline to week 6 in the scores on the BDI-1A and the SIDAS were analyzed with the use of the permutation test stratified according to baseline scores. The score at 6 weeks on the PRSexDQ was analyzed with the use of a Wilcoxon test. The score at 6 weeks on the PTCS was analyzed with the use of the Jonckheere–Terpstra trend test” (p. 1405-1406) |
| 5b. Description of approach for the handling of recurrent AEs | *“All adverse events that occurred or worsened between dosing-day 1 and week 6 were recorded and coded with the use of the Medical Dictionary for Regulatory Activities, version 23.0.” (p.1405) |
| 6a. Reported withdrawals because of AE in each arm | “In the escitalopram group, 5 of 29 patients did not complete the protocol requirements: 4 stopped taking their escitalopram capsules because of adverse events” (p. 1406). And “In the psilocybin group, 3 of 30 patients did not complete all dosing procedures: 2 missed dosing-day 2 and subsequent visits because of Covid-19–related restrictions, and 1 stopped taking daily placebo capsules after guessing their content.” (p. 1406) |
| 6b. Reported deaths and serious AEs | “No serious adverse events were observed in either trial group.” (p. 1407) |
| 7a. Provided denominators for AEs | “The percentage of patients reporting adverse events was similar in the two groups: 26 (87%) in the psilocybin group and 24 (83%) in the escitalopram group (Table 3, and Fig. S6).” (p. 1407) |
| 7b. Provided definitions used for analysis set (intention to treat, per protocol, safety data available, unclear | “Thus, 59 patients were enrolled and underwent randomization; 30 were assigned to the psilocybin group and 29 to the escitalopram group…In the escitalopram group, 5 of 29 patients did not complete the protocol requirements: 4 stopped taking their escitalopram capsules because of adverse events, and 1 missed dosing-day 2 and subsequent visits owing to restrictions related to coronavirus disease 2019 (Covid-19)… In the psilocybin group, 3 of 30 patients did not complete all dosing procedures: 2 missed dosing-day 2 and subsequent visits because of Covid-19–related restrictions, and 1 stopped taking daily placebo capsules after guessing their content.” (p. 1406) |
| 8a. Reported results separately for each treatment arm | “No serious adverse events were observed in either trial group. The percentage of patients reporting adverse events was similar in the two groups: 26 (87%) in the psilocybin group and 24 (83%) in the escitalopram group (Table 3, and  Fig. S6).” (p. 1407) |
| 8b. Severity and grading of AEs | *“No serious adverse events were observed in either trial group.” (p. 1407) |
| 8c. Provided both number of AEs and number of patients with AEs** | “The percentage of patients reporting adverse events was similar in the two  groups: 26 (87%) in the psilocybin group and 24 (83%) in the escitalopram group (Table 3, and Fig. S6).” (p. 1407) |
| 9. Described subgroup analysis and exploratory analysis for harms | Not Conducted |
| 10a. Provided a balanced view that puts benefits and harms into perspective | “In this 6-week randomized trial comparing psilocybin with escitalopram in patients with long-standing, mild-to-severe depression, the change in depression scores on the QIDS-SR-16 at week 6 (the primary outcome) did not differ significantly between the trial groups. Secondary outcomes generally favored psilocybin over escitalopram; however, the confidence intervals for the between-group differences were not adjusted for multiple comparisons, and no conclusions can be drawn from these data.” (p. 1408). And, “The incidence of adverse events was similar in the trial groups, and no serious adverse events occurred. The percentages of patients who had anxiety, dry mouth, sexual dysfunction, or reduced emotional responsiveness were higher  in the escitalopram group than in the psilocybin group.19 Four patients in the escitalopram group stopped taking their daily capsules entirely, and 1 patient halved the dose because of perceived adverse events. No patient in the psilocybin group requested to cancel the second psilocybin dose.” (p. 1408) |
| 10b. Included limitations of study with respect to harms (e.g. lack of power, short duration of exposure, inconclusive findings, post hoc analysis, generalizability of AE info as dependent on clinical setting) | “Acute subjective effects of psilocybin relating to the psychedelic experience were not included as adverse events in our trial, because previous  studies have suggested that they may have a mediating influence on positive outcomes…This requirement informed this trial’s screening criteria that excluded patients with preexisting psychiatric conditions believed to be incompatible with the limited psychological support that could be made available within the trial. This exclusion criterion may have biased the trial sample toward patients who could receive psilocybin without unacceptable side effects.” (p. 1408-1409) |
| AE=Adverse Event | |

*Note*: *Indicates that the authors mentioned information that could conceivably have met this criterion, but the information reported was insufficient. It is not included in the final tally. ** This is a double-barreled question; the authors met a portion of the criteria. It is not included in the final tally.

| Supplementary File 5 | |
| --- | --- |
| Davis et al., 2021 | |
| Criteria | Example Text w/Page Number |
| 1. AEs mentioned in title or abstract | Not Specified |
| 2. Information on AEs mentioned in the introduction | *“Moreover, psilocybin has lower addiction liability and toxic effects compared with ketamine^20-22^ and is generally not associated with long-term perceptual, cognitive, or neurological dysfunction.^23^” (p. 482) |
| 3a. Definitions of AEs mentioned | Not Specified |
| 3b. If article mentioned all or selected sample of AE | *“Secondary outcome measures for depressive symptoms were the Beck Depression Inventory II (score range: 0-63, with higher scores indicating severe depression)^40^ and the 9-item Patient Health Questionnaire (score range: 0-27, with higher scores indicating severe depression).^41^ The Columbia-Suicide Severity Rating Scale (severity of ideation subscale score range: 0-5, with higher scores indicating presence of ideation with at least some intent to die)^42,43^ was completed at every visit to assess for potentially worsening suicidal ideation throughout the trial. Anxiety symptoms were measured using the clinician-administered Hamilton Anxiety Rating Scale (score range: 0-56, with higher scores indicating severe anxiety)^44^ and the State-Trait Anxiety Index (score range: 0-80, with higher scores indicating greater anxiety).^45^  Blood pressure and heart rate were examined before and during the psilocybin sessions.” (p. 484) |
| 3c. If article mentioned the use of a validated instrument to report AEs severity | *“Secondary outcome measures for depressive symptoms were the Beck Depression Inventory II (score range: 0-63, with higher scores indicating severe depression)^40^ and the 9-item Patient Health Questionnaire (score range: 0-27, with higher scores indicating severe depression).^41^ The Columbia-Suicide Severity Rating Scale (severity of ideation subscale score range: 0-5, with higher scores indicating presence of ideation with at least some intent to die)^42,43^ was completed at every visit to assess for potentially worsening suicidal ideation throughout the trial. Anxiety symptoms were measured using the clinician-administered Hamilton Anxiety Rating Scale (score range: 0-56, with higher scores indicating severe anxiety)^44^ and the State-Trait Anxiety Index (score range: 0-80, with higher scores indicating greater anxiety).^45^  Blood pressure and heart rate were examined before and during the psilocybin sessions.” (p. 484) |
| 4a. Describe the mode of data collection (e.g. diaries, phone interviews, face-to-face interviews) | “Consistent with previous studies using psilocybin,^16,31^ the visit schedule included preparatory meetings (8 hours in total) with 2 session facilitators before the first psilocybin session as well as follow-up meetings after psilocybin sessions (2-3 hours in total) (eMethods in Supplement 2).” And “For safety during the 8-week delay period of the delayed treatment group, participants were monitored weekly by in-person assessment or brief telephone calls.” (p. 483) |
| 4b. Stated the timing of collection of AE data | “The delay interval was 8 weeks, after which participants in the delayed treatment group underwent all study assessments and entered the study intervention period. Randomization to the immediate treatment and delayed treatment groups occurred after screening and baseline assessments (Figure 1).” (p. 483). And “The intervention period was 8 weeks and involved at least 18  in-person visits, including 2 daylong psilocybin administration sessions (Figure 2).” (p. 483). And “The Columbia-Suicide Severity Rating Scale (severity of ideation subscale score range:0-5, with higher scores indicating presence of ideation with at least some intent to die) ^42,43^ was completed at every visit to assess for potentially worsening suicidal ideation throughout the trial.” (p. 484). |
| 4c. Description of how AE were attributed to trial drugs | Not Specified |
| 4d. Described the plan for monitoring for harms and rules for stopping the trial because of harms ** | *“The Columbia-Suicide Severity Rating Scale (severity of ideation subscale score range: 0-5, with higher scores indicating presence of ideation with at least some intent to die)^42,43^ was completed at every visit to assess for potentially worsening suicidal ideation throughout the trial. Anxiety symptoms were measured using the clinician-administered Hamilton Anxiety Rating Scale (score range: 0-56, with higher scores indicating severe anxiety)^44^ and the State-Trait Anxiety Index (score range: 0-80, with higher scores indicating greater anxiety).^45^ Blood pressure and heart rate were examined before and during the psilocybin sessions.” (p. 484) |
| 5a. Described the methods for presenting and/or analyzing AEs | Not Specified |
| 5b. Description of approach for the handling of recurrent AEs | Not Specified |
| 6a. Reported withdrawals because of AE in each arm | Not Specified |
| 6b. Reported deaths and serious AEs | “There were no serious adverse events in this trial.” (P. 486) |
| 7a. Provided denominators for AEs | *“Other nonserious adverse effects, which occurred during the psilocybin administration, that were reported by participants after completing at least one-half of the psilocybin sessions included challenging emotional (eg, fear and sadness) and physical (eg, feeling body shake or tremble) experiences (eTable 8 in Supplement 2). Mild to moderate transient headache was reported during 16 of 48 sessions (33%) and after the subjective psilocybin effects had subsided after 14 of 48 sessions (29%).” (p. 486) |
| 7b. Provided definitions used for analysis set (intention to treat, per protocol, safety data available, unclear | Not Specified |
| 8a. Reported results separately for each treatment arm | Not Specified |
| 8b. Severity and grading of AEs | “There were no serious adverse events in this trial. A transient increase in blood pressure that exceeded the protocol criteria for more frequent assessment (ie, diastolic blood pressure >100 mmHg) occurred during 1 session, but no medical intervention was needed, and the blood pressure level remained  within predetermined safety parameters and resolved spontaneously during the session (eTable 7 in Supplement 2).” (p. 486) And “Mild to moderate transient headache was reported during 16 of 48 sessions (33%) and after the subjective psilocybin effects had subsided after 14 of 48 sessions (29%).” (p. 486) |
| 8c. Provided both number of AEs and number of patients with AEs | Not Specified |
| 9. Described subgroup analysis and exploratory analysis for harms | Not Conducted |
| 10a. Provided a balanced view that puts benefits and harms into perspective | “The present trial showed that psilocybin administered in the context of supportive psychotherapy (approximately 11 hours) produced large, rapid, and sustained antidepressant effects. The effect sizes reported in this study were approximately 2.5 times greater than the effect sizes found in psychotherapy^57^ and more than 4 times greater than the effect sizes found in psychopharmacological depression treatment studies.^58^”… “Furthermore, given that psilocybin was associated with nonserious adverse effects that were frequently reported as mild-to-moderate head-ache and challenging emotions that were limited to the time of sessions (eTables 8 and 9 in Supplement 2), this intervention may be more acceptable to patients than widely prescribed antidepressant medications that confer substantially more problematic effects (eg, suicidal ideation, decrease in sexual drive, and weight gain).” (p. 487) |
| 10b. Included limitations of study with respect to harms (e.g. lack of power, short duration of exposure, inconclusive findings, post hoc analysis, generalizability of AE info as dependent on clinical setting) | “This study has some other limitations. It had a short-term  follow-up, a small sample that was predominantly composed of White non-Hispanic participants, and included participants with low risk of suicide and moderately severe depression. Further research with larger and more diverse samples, longer term follow-up, and a placebo control is needed to better ascertain the safety (eg, abuse potential of psilocybin, suicide risk, and emergence of psychosis) and efficacy of this intervention among patients with MDD.” (p. 487) |
| AE=Adverse Event | |

*Note*: *Indicates that the authors mentioned information that could conceivably have met this criterion, but the information reported was insufficient. It is not included in the final tally. ** This is a double-barreled question; the authors met a portion of the criteria. It is not included in the final tally.

| Supplementary File 6 | |
| --- | --- |
| Goodwin et al., 2022 | |
| Criteria | Example Text w/Page Number |
| 1. AEs mentioned in title or abstract | “Adverse events occurred in 179 of 233 participants (77%) and included headache, nausea, and dizziness. Suicidal ideation or behavior or self-injury occurred in all dose groups.” (p. 1637) |
| 2. Information on AEs mentioned in the introduction | Not Specified |
| 3a. Definitions of AEs mentioned | “Adverse events that emerged or worsened after trial-drug administration were categorized as serious adverse events on the basis of the ICH Good Clinical Practice criteria and with the use of additional information from the Columbia Suicide Severity Rating Scale.^16^ Suicidal ideation with intent or endorsement of any items in the suicidal-behavior section, including nonsuicidal self-injurious behavior, was reported as a serious adverse event.” (p. 1640) |
| 3b. If article mentioned all or selected sample of AE | “Adverse events that emerged or worsened after trial-drug administration were categorized as serious adverse events on the basis of the ICH Good Clinical Practice criteria and with the use of additional information from the Columbia Suicide Severity Rating Scale.^16^ Suicidal ideation with intent or endorsement of any items in the suicidal-behavior section, including nonsuicidal self-injurious behavior, was reported as a serious adverse event. Safety assessments also included evaluation of vital signs (at screening, baseline, day 1, and day 2), clinical laboratory tests (including urine drug screening) (at screening, day 2, and week 3), and 12-lead electrocardiography (ECG) at screening and day 2.” (p.1640) |
| 3c. If article mentioned the use of a validated instrument to report AEs severity | “Adverse events were evaluated at every visit and were recorded and coded with the use of the Medical Dictionary for Regulatory Activities (MedDRA), version 23.0. All visits were in conducted in per- son except for the week 6 and 9 visits, which were conducted remotely. Adverse events that emerged or worsened after trial-drug administration were categorized as serious adverse events on the basis of the ICH Good Clinical Practice criteria and with the use of additional information from the Columbia Suicide Severity Rating Scale.^16^” (p. 1640) |
| 4a. Describe the mode of data collection (e.g. diaries, phone interviews, face-to-face interviews) | “Adverse events were evaluated at every visit and were recorded and coded with the use of the Medical Dictionary for Regulatory Activities (MedDRA), version 23.0. All visits were in conducted in per- son except for the week 6 and 9 visits, which were conducted remotely. Adverse events that emerged or worsened after trial-drug administration were categorized as serious adverse events on the basis of the ICH Good Clinical Practice criteria and with the use of additional information from the Columbia Suicide Severity Rating Scale.^16^” (p. 1640) |
| 4b. Stated the timing of collection of AE data | “Adverse events were evaluated at every visit and were recorded and coded with the use of the Medical Dictionary for Regulatory Activities (MedDRA), version 23.0. All visits were in conducted in person except for the week 6 and 9 visits, which were conducted remotely.” And “Safety assessments also included evaluation of vital signs (at screening, baseline, day 1, and day 2), clinical laboratory tests (including urine drug screening) (at screening, day 2, and week 3), and 12-lead electrocardiography (ECG) at screening and day 2.” (p. 1640) |
| 4c. Description of how AE were attributed to trial drugs | “Adverse events that emerged or worsened after trial-drug administration were categorized as serious adverse events on the basis of the ICH Good Clinical Practice criteria and with the use of additional information from the Columbia Suicide Severity Rating Scale.^16^” (p. 1640) |
| 4d. Described the plan for monitoring for harms and rules for stopping the trial because of harms ** | “Adverse events that emerged or worsened after trial-drug administration were categorized as serious adverse events on the basis of the ICH Good Clinical  Practice criteria and with the use of additional information from the Columbia Suicide Severity Rating Scale.^16^ Suicidal ideation with intent or endorsement of any items in the suicidal-behavior section, including nonsuicidal self-injurious behavior, was reported as a serious adverse event. Safety assessments also included evaluation of vital signs (at screening, baseline, day 1, and day  2), clinical laboratory tests (including urine drug screening) (at screening, day 2, and week 3), and 12-lead electrocardiography (ECG) at screening and day 2.” (p. 1640) |
| 5a. Described the methods for presenting and/or analyzing AEs | “Descriptive statistics were used to analyze safety data from all randomly assigned participants who received single-dose treatment (safety analysis set), including adverse events, concomitant medications, evaluation of vital signs, clinical laboratory tests, findings from 12-lead ECG, and suicidality assessments.” (p.1640) |
| 5b. Description of approach for the handling of recurrent AEs | Not Specified |
| 6a. Reported withdrawals because of AE in each arm | “(Fig. 1)” (p. 1641) |
| 6b. Reported deaths and serious AEs | “Adverse events that were rated as severe on day 1 were reported by 4% of the participants in the 25-mg group, 8% of those in the 10-mg group, and 1% of those in the 1-mg group…From day 2 up to week 3 (primary end-point assessment), severe adverse events were reported by 9% of the participants in the 25-mg group, 7% of those in the 10-mg group, and 1% of those in the 1-mg group…After week 3 and up to week 12 (end of trial), severe adverse events were reported by 3% of the participants in the 25-mg group, 4% of those in the 10-mg group, and no participants in the 1-mg group.” (p. 1644) |
| 7a. Provided denominators for AEs | “Adverse events occurred in 66 participants (84%) in the 25-mg group, 56 (75%) in the 10-mg group, and 57 (72%) in the 1-mg group.” (p. 1644) |
| 7b. Provided definitions used for analysis set (intention to treat, per protocol, safety data available, unclear) | “A total of 79 participants were assigned to the 25-mg group, 75 to the 10-mg group, and 79 to the 1-mg group (Fig. 1). By week 12, a total of 5 participants (6%) in the 25-mg group, 9 (12%) in the 10-mg group, and 10 (13%) in the 1-mg group had withdrawn from the trial.” And “(Fig. 1)” (p. 1641) |
| 8a. Reported results separately for each treatment arm | “Adverse events that were rated as severe on day 1 were reported by 4% of the participants in the 25-mg group, 8% of those in the 10-mg group, and 1% of those in the 1-mg group…From day 2 up to week 3 (primary end-point assessment), severe adverse events were reported by 9% of the participants in the 25-mg group, 7% of those in the 10-mg group, and 1% of those in the 1-mg group…After week 3 and up to week 12 (end of trial), severe adverse events were reported by 3% of the participants in the 25-mg group, 4% of those in the 10-mg group, and no participants in the 1-mg group.” (p. 1644) |
| 8b. Severity and grading of AEs | “The most frequent adverse events reported in the 25-mg group with onset on the day of psilocybin administration (day 1) were headache (in 24% of the participants), nausea (in 22%), and dizziness and fatigue (in 6% each) (Table 3). Adverse events that were rated as severe on day 1 were reported by 4% of the participants in the 25-mg group, 8% of those in the 10-mg group, and 1% of those in the 1-mg group…The serious adverse events in the 25-mg group were suicidal ideation (in two participants) and intentional self-injury (nonsuicidal self-injurious behavior) (in two participants) and in the 10-mg group were suicidal ideation (in two participants), intentional self- injury (in one participant), and hospitalization (for severe depression, in one participant). No serious adverse events were reported from day 2 up to week 3 in the 1-mg group…Serious adverse events in the 25-mg group were suicidal behavior (in three participants), codeine withdrawal syndrome (in one participant), and adjustment disorder with anxiety and depressed mood (in one participant); in the 10-mg group were intentional self-injury (in one participant), depression (in one participant), and suicidal ideation (in one participant); and in the 1-mg group were intentional self-injury (in one participant). Severe adverse events during the trial period according to MedDRA system organ class and preferred term are shown in Table S7.” (p. 1644) |
| 8c. Provided both number of AEs and number of patients with AEs | “Adverse events occurred in 66 participants (84%) in the 25-mg group, 56 (75%) in the 10-mg group, and 57 (72%) in the 1-mg group. The most frequent adverse events reported in the 25-mg group with onset on the day of psilocybin administration (day 1) were headache (in 24% of the participants), nausea (in 22%), and dizziness and fatigue (in 6% each) (Table 3). Adverse events that were rated as severe on day 1 were reported by 4% of the participants in the 25-mg group, 8% of those in the 10-mg group, and 1% of those in the 1-mg group. Just one participant (in the 25-mg group) was treated with adjunctive medication (lorazepam for acute anxiety) on day 1. There were no serious adverse events reported on day 1. From day 2 up to week 3 (primary end-point  assessment), severe adverse events were reported by 9% of the participants in the 25-mg group, 7% of those in the 10-mg group, and 1% of those in the 1-mg group. The serious adverse events in the 25-mg group were suicidal ideation (in two participants) and intentional self-injury (nonsuicidal self-injurious behavior) (in two participants) and in the 10-mg group were suicidal ideation (in two participants), intentional self- injury (in one participant), and hospitalization (for severe depression, in one participant). No serious adverse events were reported from day 2 up to week 3 in the 1-mg group. After week 3 and up to week 12 (end of trial), severe adverse events were reported by 3% of the participants in the 25-mg group, 4% of those in the 10-mg group, and no participants in the 1-mg group. Serious adverse events in the 25-mg group were suicidal behavior (in three participants), codeine withdrawal syndrome (in one participant), and adjustment disorder with anxiety and depressed mood (in one participant); in the 10-mg group were intentional self-injury (in one participant), depression (in one participant), and suicidal ideation (in one participant); and in the 1-mg group were intentional self-injury (in one  participant). Severe adverse events during the trial period according to MedDRA system organ class and preferred term are shown in Table S7.” (p. 1644) |
| 9. Described subgroup analysis and exploratory analysis for harms | “Adverse events occurred in 66 participants (84%) in the 25-mg group, 56 (75%) in the 10-mg group, and 57 (72%) in the 1-mg group. The most frequent adverse events reported in the 25-mg group with onset on the day of psilocybin administration (day 1) were headache (in 24% of the participants), nausea (in 22%), and dizziness and fatigue (in 6% each) (Table 3). Adverse events that were rated as severe on day 1 were reported by 4% of the participants in the 25-mg group, 8% of those in the 10-mg group, and 1% of those in the 1-mg group. Just one participant (in the 25-mg group) was treated with adjunctive medication (lorazepam for acute anxiety) on day 1. There were no serious adverse events reported on day 1. From day 2 up to week 3 (primary end-point  assessment), severe adverse events were reported by 9% of the participants in the 25-mg group, 7% of those in the 10-mg group, and 1% of those in the 1-mg group. The serious adverse events in the 25-mg group were suicidal ideation (in two participants) and intentional self-injury (nonsuicidal self-injurious behavior) (in two participants) and in the 10-mg group were suicidal ideation (in two participants), intentional self- injury (in one participant), and hospitalization (for severe depression, in one participant). No serious adverse events were reported from day 2 up to week 3 in the 1-mg group. After week 3 and up to week 12 (end of trial), severe adverse events were reported by 3% of the participants in the 25-mg group, 4% of those in the 10-mg group, and no participants in the 1-mg group. Serious adverse events in the 25-mg group were suicidal behavior (in three participants), codeine withdrawal syndrome (in one participant), and adjustment disorder with anxiety and depressed mood (in one participant); in the 10-mg group were intentional self-injury (in one participant), depression (in one participant), and suicidal ideation (in one participant); and in the 1-mg group were intentional self-injury (in one  participant). Severe adverse events during the trial period according to MedDRA system organ class and preferred term are shown in Table S7.” (p. 1644) |
| 10a. Provided a balanced view that puts benefits and harms into perspective | “This phase 2 clinical trial showed the feasibility of psilocybin monotherapy for up to 12 weeks in patients with a treatment-resistant episode of major depression. The change from baseline to week 3 in the MADRS total score (primary end point) was significantly better with a 25-mg dose than with a 1-mg dose; there was not significant difference between the 10-mg dose and the 1-mg dose. In addition to headache, nausea, dizziness, and fatigue, some participants had suicidal ideation or self-injurious behavior, and the proportions of these participants were numerically higher in the 25-mg and 10-mg groups than in the 1-mg group. In view of the participants who showed worsening of suicidal state, suicidality demands clinical vigilance in future trials of psilocybin for depression.” (p. 1645) |
| 10b. Included limitations of study with respect to harms (e.g. lack of power, short duration of exposure, inconclusive findings, post hoc analysis, generalizability of AE info as dependent on clinical setting) | “Limitations of the current trial include the lack of an active comparator, the lack of an ethnically diverse participant sample, and the exclusion of persons judged to be at a clinically significant risk for suicide.” And “In this trial of psilocybin administered in a single session with psychological support, a 25-mg dose but not a 10-mg dose resulted in a significantly greater reduction (improvement) in MADRS total scores than a 1-mg dose at 3 weeks in participants with treatment-resistant depression but was associated with adverse events.” (p. 1645) |
| AE=Adverse Event | |

*Note*: *Indicates that the authors mentioned information that could conceivably have met this criterion, but the information reported was insufficient. It is not included in the final tally. ** This is a double-barreled question; the authors met a portion of the criteria. It is not included in the final tally.

.

| Supplementary File 7 | |
| --- | --- |
| Marschall et al., 2021 | |
| Criteria | Example Text w/Page Number |
| 1. AEs mentioned in title or abstract | Not Specified |
| 2. Information on AEs mentioned in the introduction | Not Specified |
| 3a. Definitions of AEs mentioned | Not Specified |
| 3b. If article mentioned all or selected sample of AE | Not Specified |
| 3c. If article mentioned the use of a validated instrument to report AEs severity | Not Specified |
| 4a. Describe the mode of data collection (e.g. diaries, phone interviews, face-to-face interviews) | Not Specified |
| 4b. Stated the timing of collection of AE data | Not Specified |
| 4c. Description of how AE were attributed to trial drugs | Not Specified |
| 4d. Described the plan for monitoring for harms and rules for stopping the trial because of harms | Not Specified |
| 5a. Described the methods for presenting and/or analyzing AEs | Not Specified |
| 5b. Description of approach for the handling of recurrent AEs | Not Specified |
| 6a. Reported withdrawals because of AE in each arm | Not Specified |
| 6b. Reported deaths and serious AEs | Not Specified |
| 7a. Provided denominators for AEs | Not Specified |
| 7b. Provided definitions used for analysis set (intention to treat, per protocol, safety data available, unclear | Not Specified |
| 8a. Reported results separately for each treatment arm | *Partial information in tables 11, 12, and 13 |
| 8b. Severity and grading of AEs | Not Specified |
| 8c. Provided both number of AEs and number of patients with AEs | *Partial information in tables 12 and 13 |
| 9. Described subgroup analysis and exploratory analysis for harms | Not Specified |
| 10a. Provided a balanced view that puts benefits and harms into perspective | Not Specified |
| 10b. Included limitations of study with respect to harms (e.g. lack of power, short duration of exposure, inconclusive findings, post hoc analysis, generalizability of AE info as dependent on clinical setting) | Not Specified |
| AE=Adverse Event | |

*Note*: *Indicates that the authors mentioned information that could conceivably have met this criterion, but the information reported was insufficient. It is not included in the final tally. ** This is a double-barreled question; the authors met a portion of the criteria. It is not included in the final tally.

| Supplementary File 8 | |
| --- | --- |
| Raison et al., 2023 | |
| Criteria | Example Text w/Page Number |
| 1. AEs mentioned in title or abstract | “There were no serious treatment-emergent AEs; however, psilocybin treatment was associated with a higher rate of overall AEs and a higher rate of severe AEs” (p. 843) |
| 2. Information on AEs mentioned in the introduction | “However, recent critiques highlight notable limitations in many of these studies, 16-19 including small sample sizes, assessments by raters likely to be functionally unblinded, an open or waitlist comparator design, and an inadequate assessment of adverse events (AEs)” (p. 844). |
| 3a. Definitions of AEs mentioned | *“Treatment-emergent AEs (TEAEs) were considered any AE that occurred after drug administration. Among TEAEs, an AE was classified as “related” if there was a reasonable possibility that the study drug caused the event as judged by site principal investigators.” (p. 845) |
| 3b. If article mentioned all or selected sample of AE | “Solicited adverse events included (1) active suicidal ideation assessed with the Columbia Suicide Severity Rating Scale (C-SSRS) administered by site personnel or MADRS item 10 and verified by clinical assessment, (2) elevated blood pressure or heart rate requiring medication, (3) drug overdose with suicidal intent, (4) headache, (5) nausea, and (6) visual perceptual effects.” (p.845) |
| 3c. If article mentioned the use of a validated instrument to report AEs severity | “Serious AEs were classified as those resulting in any of a list of negative health outcomes (eg, death, inpatient hospitalization, significant or persistent incapacity, congenital birth defect/abnormality) following the standard definition.^30^” (p. 845) |
| 4a. Describe the mode of data collection (e.g. diaries, phone interviews, face-to-face interviews) | Not Specified |
| 4b. Stated the timing of collection of AE data | “Study drug administration occurred on the same day as randomization (day 1), no later than 7 days following the baseline assessment. Postdosing assessments were conducted on days 2, 8, 15, 29, and 43.” And “AEs were collected from enrollment through the end of study” (p. 845) |
| 4c. Description of how AE were attributed to trial drugs | “Among TEAEs, an AE was classified as “related” if there was a reasonable possibility that the study drug caused the event as judged by site principal investigators” (p, 845) |
| 4d. Described the plan for monitoring for harms and rules for stopping the trial because of harms** | “AEs were collected from enrollment through the end of study and were graded for severity, seriousness, and relationship to study product by site principal investigators. Treatment-emergent AEs (TEAEs) were considered any AE that occurred after drug administration.” (p. 845) |
| 5a. Described the methods for presenting and/or analyzing AEs | “Incidence of AEs was summarized using counts, percentages, and Clopper-Pearson 95% CIs for the following protocol-defined study periods: enrollment through day 43, dosing (day 1) through day 9, and day 10 through day 43. Relative incidence for TEAEs was calculated by dividing the percentage of participants experiencing an event in the psilocybin treatment group by the percentage in the niacin group and were presented with Wald 95% CIs. Percent difference in AEs between groups with 95% CIs were also calculated.” (p. 846) |
| 5b. Description of approach for the handling of recurrent AEs | Not Specified |
| 6a. Reported withdrawals because of AE in each arm | “No participants withdrew due to an AE” (p.847) |
| 6b. Reported deaths and serious AEs | “Severe solicited events were reported by 3 participants receiving psilocybin (2 headaches and 1 visual perceptual effects) and 1 headache was reported in a participant receiving niacin.” (p. 849) |
| 7a. Provided denominators for AEs | Example: “The most common solicited AE was headache in 33 of 50 participants” (p. 849) |
| 7b. Provided definitions used for analysis set (intention to treat, per protocol, safety data available, unclear | See Figure 1, example in text: “104 were randomized, received the study drug, and comprised the ITT population (51 in the psilocybin group and 53 in the niacin group)” (p. 847) |
| 8a. Reported results separately for each treatment arm | “From randomization on the day of dosing (day 1) through day 9, a total of 41 of 50 participants (82%) in the psilocybin group experienced at least 1 drug-related TEAE vs 24 of 54 (44%) in the niacin group (difference, 38% [95% CI, 20.6%-41.3%]; relative incidence [RI], 1.8 [95% CI, 1.3-1.8])” (p. 848)  See Table 3 (p. 850) |
| 8b. Severity and grading of AEs | “Three serious AEs occurred between enrollment and randomization (nephrolithiasis; incisional hernia, obstructive; and appendicitis; Table 3)” (p. 848) |
| 8c. Provided both number of AEs and number of patients with AEs | See Table 3 (p. 850) |
| 9. Described subgroup analysis and exploratory analysis for harms | See Table 3 (p. 850) Also, “In the safety population (Figure 1), 44 of 50 participants (88%) receiving psilocybin and 33 of 54 (61%) receiving niacin reported at least 1 AE through day 43. Three serious AEs occurred between enrollment and randomization (nephrolithiasis; incisional hernia, obstructive; and appendicitis; Table 3). From randomization on the day of dosing (day 1) through day 9, a total of 41 of 50 participants (82%) in the psilocybin group experienced at least 1 drug-related TEAE vs 24 of 54 (44%) in the niacin group (difference, 38% [95% CI, 20.6%-41.3%]; relative incidence [RI], 1.8 [95% CI, 1.3-1.8]). Severe related AEs through day 9 were reported by 4 of 50 participants (8%) receiving psilocybin (migraine in 1 participant, headache in 1 participant, illusion in 1 participant [all solicited], and panic attack and paranoia in 1 participant) vs 0 in the niacin group. The rates of mild and moderate drug-related TEAEs in the same period were higher for psilocybin vs niacin (mild: difference, 35% [95% CI, 17.9%-52.9%]; RI, 1.8 [95% CI, 1.1-1.8]); moderate: difference, 18% [95% CI, 5.8%-30.8%]; RI, 2.3 [95% CI, 1.0-5.7]). From day 10 through day 43, related TEAEs were reported by 2 of 50 participants (4%) in the psilocybin group vs 1 of 53 (2%) receiving niacin (difference, 2% [95% CI, −4.4% to 8.7%]; RI, 2.1 [95% CI, 0.2-22.7]) (Table 3).” (p. 848) |
| 10a. Provided a balanced view that puts benefits and harms into perspective | “Psilocybin was generally well-tolerated, with most AEs being of mild or moderate severity and generally limited to the acute dosing period. The 8% rate of severe adverse events in participants receiving psilocybin was similar to the 10% rate reported in the study by Goodwin et al in participants with TRD treated with a single 25-mg dose of psilocybin.^14^ However, in contradistinction to the study by Goodwin et al, no clinically confirmed active suicidal ideation or suicidal behavior occurred in either randomized group. No serious TEAEs were reported in the current study; however, psilocybin treatment was associated with a higher rate of overall AEs and a higher rate of severe AEs compared with niacin, with these severe AEs being known effects of psilocybin.^17^ Moreover, psychedelics may produce AEs not captured by standard rating scales or may induce unrecognized new psychiatric conditions even as they improve target syndromes.^32^” (p. 850) |
| 10b. Included limitations of study with respect to harms (e.g. lack of power, short duration of exposure, inconclusive findings, post hoc analysis, generalizability of AE info as dependent on clinical setting) | “All study facilitators received extensive training; however, fidelity to the psychological support protocol by study facilitators was not assessed, leaving open the possibility that at least some degree of between-participant variability in response may be attributable to unknown differences in psychological support provided by facilitators, rather than direct biological effects of psilocybin per se. This study limitation highlights the importance of better understanding potential benefits and harms that may be engendered by the psychological/psychotherapeutic components of psilocybin assisted therapy” and “Fifth, as with the majority of recent clinical trials of psychedelics,^35^ a major limitation of the current study is a lack of participant ethnic and racial diversity. The current study sample was predominantly White, non-Hispanic, and from upper socioeconomic echelons. Whether psilocybin would be more, less, or equivalently effective in a more ethnically, racially, and socioeconomically diverse sample is an urgent question that must be addressed in future studies by actively employing strategies shown to increase recruitment and retention of racial and ethnic minoritized populations and other underrepresented groups in clinical trials of psychedelic agents.^36^ “ (p. 851). |
| AE=Adverse Event | |

*Note*: *Indicates that the authors mentioned information that could conceivably have met this criterion, but the information reported was insufficient. It is not included in the final tally. ** This is a double-barreled question; the authors met a portion of the criteria. It is not included in the final tally.

| Supplementary File 9 | |
| --- | --- |
| Rosenblat et al., 2024 | |
| Criteria | Example Text w/Page Number |
| 1. AEs mentioned in title or abstract | “Adverse events were transient, with no serious adverse events.” (p. 190) |
| 2. Information on AEs mentioned in the introduction | Not Specified |
| 3a. Definitions of AEs mentioned | Not Specified |
| 3b. If article mentioned all or selected sample of AE | “Safety and Tolerability: standard adverse event reporting, Columbia Suicidality Scale for treatment emergent-suicidality (C-SSRS), Young Mania Rating Scale (YMRS), Clinician-Administered Dissociative States Scale (CADSS), 23-item; Brief Psychiatric Rating Scale (BPRS), Mystical Experiences Questionnaire (MEQ);” (p. e4) |
| 3c. If article mentioned the use of a validated instrument to report AEs severity | *“Safety and Tolerability: standard adverse event reporting, Columbia Suicidality Scale for treatment emergent-suicidality (C-SSRS), Young Mania Rating Scale (YMRS), Clinician-Administered Dissociative States Scale (CADSS), 23-item; Brief Psychiatric Rating Scale (BPRS), Mystical Experiences Questionnaire (MEQ);” (p. e4) |
| 4a. Describe the mode of data collection (e.g. diaries, phone interviews, face-to-face interviews) | “Participants were assessed in person or virtually for all study visits and psychotherapy sessions. Follow-up assessments without repeat dose administration at weeks 1, 3, 4, 5, 6, 8, 10, 12, 14, 16, 18, 20, 22, and 24 could be conducted virtually or by telephone if required. All psilocybin administration visits were conducted on site (no virtual option). Psychotherapy sessions were conducted on site or virtually with a preference toward in person therapy whenever possible.” (p. e4) |
| 4b. Stated the timing of collection of AE data | Figure 1 (p.192) and “After the acute effects of the psilocybin had resolved, safety assessments were completed by designated study personnel.” (p. e4) |
| 4c. Description of how AE were attributed to trial drugs | “After the acute effects of the psilocybin had resolved, safety assessments were completed by designated study personnel.” And “Three sets of outcome measures were collected at the time points shown in Figure 1: (1) Safety and Tolerability: standard adverse event reporting, Columbia Suicidality Scale for treatment emergent-suicidality (C-SSRS), Young Mania Rating Scale (YMRS), Clinician-Administered Dissociative States Scale (CADSS), 23-item; Brief Psychiatric Rating Scale (BPRS), Mystical Experiences Questionnaire (MEQ)” (p.e4) and “In order to evaluate safety and feasibility, descriptive statistics of recruitment rates, retention rates and adverse events were reported.” (p.e5) |
| 4d. Described the plan for monitoring for harms and rules for stopping the trial because of harms ** | “After the acute effects of the psilocybin had resolved, safety assessments were completed by designated study personnel.” (p. e5) |
| 5a. Described the methods for presenting and/or analyzing AEs | “As a feasibility study, the primary outcome was based on meeting the following a priori feasibility criteria: (1) Less than 30% all-cause drop out before the primary week-2 endpoint, (2) zero or minimal worsening of baseline suicidality post-dose, (3) less than 15% experiencing serious adverse events (SAEs) and (4) greater than 80% of adverse events resolving within 48 h of each dose administration.” And “In order to evaluate safety and feasibility, descriptive statistics of recruitment rates, retention rates and adverse events were reported.” (p.e5) |
| 5b. Description of approach for the handling of recurrent AEs | “…participants were potentially eligible for a second psilocybin dose (accompanied by the same therapy protocol as the first dose). Eligibility for repeat doses was determined by the study doctor, based on clinical evidence of all three of the following (1) clinical benefits from the prior dose (either based on scales or clinical judgment), (2) adequate tolerability/safety of prior dose(s) and (3) signs or symptoms of relapse of depression lasting two weeks or longer.” And “. Follow-up assessments without repeat dose administration at weeks 1, 3, 4, 5, 6, 8, 10, 12, 14, 16, 18, 20, 22, and 24 could be conducted virtually or by telephone if required” (p.e4) See Figure 1 (p. 192) for a detailed account of assessing AEs repeatedly. Due to the nature of the study being feasibility their handling of AEs is mostly descriptive, although “Therapists were present for safety and support for the entire duration of the dosing session; however, they attempted to minimise interruptions to allow participants to focus internally during the psychedelic experience.” (p.e4) |
| 6a. Reported withdrawals because of AE in each arm | Not Specified |
| 6b. Reported deaths and serious AEs | “While there were a large number of adverse events reported, these were considered mild to moderate and mostly expected, with no SAEs” (p. 195) |
| 7a. Provided denominators for AEs | Not Specified |
| 7b. Provided definitions used for analysis set (intention to treat, per protocol, safety data available, unclear) | “Participants were recruited and enrolled from November 1, 2021, to February 1, 2023, with all study visits completed by July 26, 2023. As summarized in Figure 2, 135 patients were assessed for eligibility, with 31 eligible, enrolled, and randomized. One participant withdrew from the study before receiving the intervention due to challenges with tapering off antidepressant medications, leaving 16 participants randomized to immediate treatment and 14 to delayed treatment. One participant dropped out before the week-2 primary endpoint, with 29 included in the primary outcome analysis. During the 6-month follow-up period, 8 additional participants dropped out to begin other treatment, with a total of 21 participants retained for the entire 6-month follow-up period.” (p. 192) |
| 8a. Reported results separately for each treatment arm | Not Specified |
| 8b. Severity and grading of AEs | *“While there were a large number of adverse events reported, these were considered mild to moderate and mostly expected, with no SAEs. Adverse events were transient, resolving within 48 h of each dose…” (p. 195). |
| 8c. Provided both number of AEs and number of patients with AEs | Not Specified |
| 9. Described subgroup analysis and exploratory analysis for harms | Not Conducted |
| 10a. Provided a balanced view that puts benefits and harms into perspective | “Notably, while clinically and statistically significant antidepressant effects were observed in our trial, the reduction on the MADRS was lower compared to previous studies.^3^ The complexity of illness in persons enrolled in our clinical trial may account for the differential efficacy compared to studies on psilocybin with more restrictive eligibility criteria. Nevertheless, clinically significant benefits were still observed without any SAEs, suggesting that further evaluation in this group would be feasible and warranted.” (p. 196)  “As the first RCT to include BDII, the feasibility of including this group was also observed. Further, no treatment-emergent mania, hypomania, or psychosis was observed. Nevertheless, conclusions regarding safety cannot be drawn from such a small subsample (n = 4). We also included participants with comorbid personality disorders (n = 9) who have been largely excluded from previous trials. No additional safety concerns were observed in this group. Nevertheless, we recognize that the suggestion of including patients with borderline personality disorder and BD in PAP trials remains controversial, with some expert opinion advising against inclusion of these patients due to theoretical safety concerns.^1,22^ Of note, these suggestions are based on opinion rather than on empirical evidence supporting elevated safety concerns in this population.” (pp 196-197)  The present trial established feasibility for this intuitive approach of providing additional doses upon relapse as long as antidepressant effects were observed and there were no additional adverse events or safety concerns that would contraindicate a repeat dose of psilocybin.” (p. 167) |
| 10b. Included limitations of study with respect to harms (e.g. lack of power, short duration of exposure, inconclusive findings, post hoc analysis, generalizability of AE info as dependent on clinical setting) | *“There are several major limitations of the present study. The open-label design, small sample size, and use of waitlist controls (rather than a placebo-control arm) are the most significant limitations that may bias the results in favor of larger antidepressant effect sizes. The heterogeneity of the sample is both a strength and a weakness that may also impact the observed effects.” (p.197) |
| AE=Adverse Event | |

*Note*: *Indicates that the authors mentioned information that could conceivably have met this criterion, but the information reported was insufficient. It is not included in the final tally. ** This is a double-barreled question; the authors met a portion of the criteria. It is not included in the final tally.

| Supplementary File 10 | |
| --- | --- |
| Ross et al., 2016 | |
| Criteria | Example Text w/Page Number |
| 1. AEs mentioned in title or abstract | Not Specified |
| 2. Information on AEs mentioned in the introduction | Not Specified |
| 3a. Definitions of AEs mentioned | Not Specified |
| 3b. If article mentioned all or selected sample of AE | Not Specified |
| 3c. If article mentioned the use of a validated instrument to report AEs severity | *“Adverse events (AEs) attributed to study  medications (psilocybin, niacin) were monitored throughout the  trial, including during and after medication administration  sessions. Systolic and diastolic blood pressure (BP) and heart rate (HR) were measured at the following time points during the medication dosing sessions: baseline, 30, 60, 90, 120, 180, 240, 300, 360 minutes post-dose administration” (p. 1167) |
| 4a. Describe the mode of data collection (e.g. diaries, phone interviews, face-to-face interviews) | Not Specified |
| 4b. Stated the timing of collection of AE data | See Figure 2 (p. 1168) |
| 4c. Description of how AE were attributed to trial drugs | “Adverse events (AEs) attributed to study medications (psilocybin, niacin) were monitored throughout the trial, including during and after medication administration sessions.” (p. 1167) |
| 4d. Described the plan for monitoring for harms and rules for stopping the trial because of harms | Not Specified |
| 5a. Described the methods for presenting and/or analyzing AEs | “For cardiovascular measures assessed during the medication sessions, repeated measures regressions, from the mixed effect repeat measurement (MMRM) model, were conducted in SAS PROC MIXED using an AR(1) covariance structure and fixed effects of time, drug (psilocybin vs. niacin) and group (niacin first vs. psilocybin first) collapsed across treatment order at time points: baseline, 30, 60, 90, 120, 180, 240, 300, 360 post-dosing (Supplementary Figure 1)” (p. 1171) |
| 5b. Description of approach for the handling of recurrent AEs | Not Specified |
| 6a. Reported withdrawals because of AE in each arm | “There were no serious AEs, either medical or psychiatric, in the trial that were attributed to either psilocybin or niacin. Regarding psychiatric AEs, no pharmacological interventions (e.g. benzodiazepines, anti-psychotics) were needed during dosing sessions, no participants abused or became addicted to psilocybin, there were no cases of prolonged psychosis or hallucinogen persisting perceptual disorder (HPPD), and no participants required psychiatric hospitalization.” (p. 1173)  Additionally, Figure 1 (p. 1167) indicates all withdrawals, none related to AEs. |
| 6b. Reported deaths and serious AEs | “There were no serious AEs, either medical or psychiatric, in the trial that were attributed to either psilocybin or niacin.” (p. 1173)  Further, Figure 1 (p. 1167) indicates all deaths that occurred during the trial |
| 7a. Provided denominators for AEs | Not Specified |
| 7b. Provided definitions used for analysis set (intention to treat, per protocol, safety data available, unclear | Not Specified |
| 8a. Reported results separately for each treatment arm | *“There were no serious AEs, either medical or psychiatric, in the trial that were attributed to either psilocybin or niacin.” (p.1173). “Cardiovascular effects during dosing sessions. Compared to the control, psilocybin produced statistically significant differences in the following cardiovascular measures and time points: systolic BP: 60, 90, 120, 180, 240, 300 minutes; diastolic BP 60, 90, 120, 180 minutes; pulse: 90, 120 minutes (see Supplementary Figure 1). Cardiovascular effects with psilocybin generally peaked at 180 minutes post-dosing and decreased towards pre-drug levels over the remainder of the session. Regarding the psilocybin first group, peak mean systolic and diastolic BPs were 142/83 (both recorded at 180 minutes post-dosing), while peak mean HR for this group was 71 at 300 minutes post-dosing (see Supplementary Figure 1).” (p.1173 – 1174) |
| 8b. Severity and grading of AEs | “The medical AEs (non-clinically significant elevations in BP and HR, headaches, nausea), and psychiatric AEs (transient anxiety, transient near-psychotic symptoms) attributable to psilocybin are all known AEs of psilocybin, were transient, tolerable, and consistent with prior trials of psilocybin administration in normal volunteers (Griffiths et al., 2006, 2008, 2011), and patients with terminal cancer (Grob et al., 2011).” (p. 1173) |
| 8c. Provided both number of AEs and number of patients with AEs | Not Specified |
| 9. Described subgroup analysis and exploratory analysis for harms | Not Reported |
| 10a. Provided a balanced view that puts benefits and harms into perspective | “There were no serious AEs, either medical or psychiatric, in the trial that were attributed to psilocybin. Since the early 1990s, approximately 2000 doses of psilocybin (ranging from low to high doses) have been safely administered to humans in the United States and Europe, in carefully controlled scientific settings, with no reports of any medical or psychiatric serious AEs, including no reported cases of prolonged psychosis or HPPD (Studerus et al., 2011). This finding is consistent with a US population (2001–2004 data from the National Survey on Drug Use and Health) based study that found no associations between lifetime use of any of the serotoninergic psychedelics (including psilocybin) and increased rates of mental illness (Krebs and Johansen, 2013). It is important to monitor closely for the emergence of transient difficult psychological states (e.g. anxiety, paranoia) in these trials and to manage them. Difficult experiences are not necessarily pathological and can be understood as part of the therapeutic process (e.g. working through cancer-related psychological or existential distress through challenging encounters or emotionally charged confrontations with cancer-related fearful imagery or symbolism) (Carbonaro et al., 2016).” (p. 1176) |
| 10b. Included limitations of study with respect to harms (e.g. lack of power, short duration of exposure, inconclusive findings, post hoc analysis, generalizability of AE info as dependent on clinical setting) | “This trial was limited by a relatively small sample size, a non-nationally representative cancer patient population (e.g. 62% women, 90% Caucasian), which decreases generalizability, a crossover design that limited the interpretation of clinical benefits after the crossover, and the use of a control with limited blinding.” (p. 1176)  “Further empirical research is needed definitively to establish its safety and efficacy.” (p. 1177) |
| AE=Adverse Event | |

*Note*: *Indicates that the authors mentioned information that could conceivably have met this criterion, but the information reported was insufficient. It is not included in the final tally. ** This is a double-barreled question; the authors met a portion of the criteria. It is not included in the final tally.

| Supplementary File 11 | |
| --- | --- |
| von Rotz et al., 2023 | |
| Criteria | Example Text w/Page Number |
| 1. AEs mentioned in title or abstract | “No serious adverse events were recorded” (p. 1) |
| 2. Information on AEs mentioned in the introduction | Not Specified |
| 3a. Definitions of AEs mentioned | *“In case of emergency during acute drug effects nifedipine (10 mg) for hypertension, diazepam (5–10 mg) for severe anxiety and olanzapine (5–10 mg) were prepared for every administration day. Study staff was instructed to attenuate adverse reactions by psychological support before administering rescue medication. No rescue medication was used during the course of the trial.” (p. 5)  “Suicidal ideation is derived from the intensity of ideation subscale from the Colombia Suicidality Severity Rating Scale (C-SSRS) assessing suicidal thoughts with a scale ranging from zero to whereas scores greater than three imply acute intentionality.” (p. 4) [note that this is included in Table 1; however, Table 1 is reference in the Method section, and Table 1 is displayed within the Method section]  “Reporting of AEs comprised clinically relevant symptoms outlasting acute drug effects, i.e., excluding transient adverse symptoms caused by psilocybin which are described in detail elsewhere.” (p. 5) |
| 3b. If article mentioned all or selected sample of AE | Not Specified |
| 3c. If article mentioned the use of a validated instrument to report AEs severity | *“Tolerability of acute drug effects was monitored by hourly evaluations of blood pressure and pulse. Rate-pressure products enabling quantification of hemodynamic response were obtained by multiplying systolic blood pressure with heart rate.” (p. 5)  “The Colombia - Suicidality Severity Rating Scale (C-SSRS) was used for the quantification of suicidal ideation and early detection of potential suicidal behaviour.” (p. 5) |
| 4a. Describe the mode of data collection (e.g. diaries, phone interviews, face-to-face interviews) | “To ensure adequate monitoring of safety-related parameters, the following endpoints were assessed at each study visit: Adverse events (AEs), psychological and physical well-being, suicidality, vital signs, and the use of concomitant medication. Tolerability of acute drug effects was monitored by hourly evaluations of blood pressure and pulse. Rate-pressure products enabling quantification of hemodynamic response were obtained by multiplying systolic blood pressure with heart rate.^16^ Reporting of AEs comprised clinically relevant symptoms outlasting acute drug effects, i.e., excluding transient adverse symptoms caused by psilocybin which are described in detail elsewhere.^4,10^” |
| 4b. Stated the timing of collection of AE data | “To ensure adequate monitoring of safety-related parameters, the following endpoints were assessed at each study visit: Adverse events (AEs), psychological and physical well-being, suicidality, vital signs, and the use of concomitant medication.” (p.5)  “Tolerability of acute drug effects was monitored by hourly evaluations of blood pressure and pulse.” (p. 5) |
| 4c. Description of how AE were attributed to trial drugs | *“Reporting of AEs comprised clinically relevant symptoms outlasting acute drug effects, i.e., excluding transient adverse symptoms caused by psilocybin which are described in detail elsewhere.^4^” (p.5) |
| 4d. Described the plan for monitoring for harms and rules for stopping the trial because of harms ** | *“To ensure adequate monitoring of safety-related parameters, the following endpoints were assessed at each study visit: Adverse events (AEs), psychological and physical well-being, suicidality, vital signs, and the use of concomitant medication.” (p.5)  “In case of emergency during acute drug effects nifedipine (10 mg) for hypertension, diazepam (5–10 mg) for severe anxiety and olanzapine (5–10 mg) were prepared for every administration day. Study staff was instructed to attenuate adverse reactions by psychological support before administering rescue medication.” (p. 5) |
| 5a. Described the methods for presenting and/or analyzing AEs | Not Specified |
| 5b. Description of approach for the handling of recurrent AEs | Not Specified |
| 6a. Reported withdrawals because of AE in each arm | “Three additional participants withdrew from further participation: one participant due to Covid-19 related issues (psilocybin group) and two (one in each group) wanted to get back on antidepressant medication resulting in exclusion from the trial. Missing values of these participants were imputed as the last observation carried forward for the primary efficacy analyses (see Fig. 1).” (p. 5–6) |
| 6b. Reported deaths and serious AEs | “No rescue medication was used during the course of the trial. No events of symptomatic hypertension, extreme anxiety and psychotic/delusional decompensation were documented. A total of 11 adverse events (i.e., clinically relevant symptoms outlasting acute drug effects) were recorded (Supplemental Table S2). All four cases of headache and the two cases of dizziness were categorised as “likely related” due to the temporal relationship to the intervention. All cases were reported to be mild. The case of diarrhea was already prevalent one day before drug administration, slightly intensified and continued thereafter and was thus categorised as “probably related”. The two cases of common cold and the case of cystitis were classified as “unlikely to be related” to the intervention.” (p.7) |
| 7a. Provided denominators for AEs | Not Specified |
| 7b. Provided definitions used for analysis set (intention to treat, per protocol, safety data available, unclear | Not Specified |
| 8a. Reported results separately for each treatment arm | *“Cardiovascular safety endpoints consisted of: 1) heart rate, 2) systolic and diastolic blood pressure [mmHg], and 3) rate pressure product (RPP). Heart rate was not different between the conditions throughout the 7 h of assessment P > 0.05. Mean systolic and diastolic blood pressure were highest 60 min post-intake (+14.5 mmHg; t(41.6) =−2.96; CI 4.6–24.4; P = 0.0051 for systolic blood pressure and +12.5 mmHg; t(40.1) =−5.09; CI 6.1–18.9; P = 0.0003 for diastolic blood pressure in the psilocybin condition. Systolic blood pressure was statistically different from baseline (30 min pre-intake) up to 5 h post-intake while diastolic blood pressure was significantly higher than at baseline up to 4 h after drug administration in the psilocybin condition. RPP was significantly higher than baseline 60 min after administration in the psilocybin condition compared to placebo: t(45.9) =−2.39; CI−2638 to−244; P = 0.021.” (p. 7).  “No rescue medication was used during the course of the trial. No events of symptomatic hypertension, extreme anxiety and psychotic/delusional decompensation were documented. A total of 11 adverse events (i.e., clinically relevant symptoms outlasting acute drug effects) were recorded (Supplemental Table S2). All four cases of headache and the two cases of dizziness were categorised as“likely related” due to the temporal relationship to the intervention. All cases were reported to be mild. The case of diarrhea was already prevalent one day before drug administration, slightly intensified and continued thereafter and was thus categorised as “probably related”. The two cases of common cold and the case of cystitis were classified as“unlikely to be related” to the intervention.” (p. 7) |
| 8b. Severity and grading of AEs | *“No events of symptomatic hypertension, extreme anxiety and psychotic/delusional decompensation were documented.” (p. 7)  “All four cases of headache and the two cases of dizziness were categorised as “likely related” due to the temporal relationship to the intervention. All cases were reported to be mild.” (p. 7) |
| 8c. Provided both number of AEs and number of patients with AEs | Not Specified |
| 9. Described subgroup analysis and exploratory analysis for harms | Not Conducted |
| 10a. Provided a balanced view that puts benefits and harms into perspective | “At the dose tested, psilocybin-assisted therapy produced only moderate and transient cardiostimulant effects, as reported previously in dose–response studies using equivalent doses.^21,22^ In fact, psilocybin increased systolic and diastolic blood pressure over 5 h, but the heart rate and the rate pressure product as a measure of cardiovascular challenge did not differ statistically from placebo. Hence, despite the possibility of a short-lasting moderate elevation of blood pressure, in participants without pre-existing medical conditions, no cardiovascular complications must be expected from administration of psilocybin in moderate dosages. Moreover, in the present trial, psilocybin was found to have a mild adverse event profile. Of a total of eight adverse events, the most frequently reported was mild headache (11%) which resolved completely within two days after drug administration. The incidence of headache in this study was substantially lower than the rate for mild to moderate headache (24–60%) reported in other clinical trials in MDD using higher doses of psilocybin.^4,6,8,9^ To what extent the incidence of headache depends on dose and/or other psychological and physiological factors needs further investigation. In the present study, suicidal ideation, including potential emergence of suicidal behaviour was assessed by a trained clinician at each study visit. No cases of suicidal behaviour occurred during the trial period of approximately one month. Moreover, at 2-week post-treatment, the scores for suicidal ideation in the psilocybin group corresponded to no suicidal ideation in 24/26 participants, while in the placebo condition, 7/26 reported scores ≥1. The present findings suggest that a moderate dose of psilocybin in a clinical setting with psychological support may not increase the risk for suicidal behaviours in pre-screened patients not displaying acute” (pp .9–10) |
| 10b. Included limitations of study with respect to harms (e.g. lack of power, short duration of exposure, inconclusive findings, post hoc analysis, generalizability of AE info as dependent on clinical setting) | Not Specified |
| AE=Adverse Event | |

*Note*: *Indicates that the authors mentioned information that could conceivably have met this criterion, but the information reported was insufficient. It is not included in the final tally. ** This is a double-barreled question; the authors met a portion of the criteria. It is not included in the final tally.
