# Supplementary material for: Incremental efficacy systematic review and meta-analysis of psilocybin-for-depression RCTs
Source: Psychopharmacology (Berl). 2025 Apr 23;242(10):2139–57. doi: 10.1007/s00213-025-06788-w (PMC12449434; doi:10.1007/s00213-025-06788-w)
Supplement: Supplementary file 5 — Supplementary file5 (DOCX 40 KB) [file 213_2025_6788_MOESM5_ESM.docx]

**Supplementary File 13**

Identified mechanisms of action by study.

| **Study ID** | **Mechanism of Action** | **Intro. vs. Discussion** | **Quote** | **Page Num.** | **Level of Analysis: Neurophysiological** | | **Level of Analysis: Psychological** | |  |
| --- | --- | --- | --- | --- | --- | --- | --- | --- | --- |
| Back et al., 2024 | Therapeutic Intervention (PAT) | Introduction, Discussion | “In earlier studies, psilocybin administered in the context of psychological support or therapy demonstrated improvements in individuals with major depressive disorder and treatment-resistant depression. Psilocybin therapy has also improved symptoms of depression and anxiety in patients with cancer, whose symptoms also followed a life event and involved confrontations with mortality. In this randomized clinical trial, we aimed to investigate whether psilocybin therapy could improve symptoms of depression, burnout, and PTSD in US clinicians who developed these symptoms from frontline clinical work during the pandemic.”; “To our knowledge, this trial is the first to demonstrate the utility of psilocybin therapy in the treatment of physicians, APPs, and nurses who developed moderate to severe symptoms of depression in the course of frontline work during the COVID-19 pandemic.”; “This trial builds on past studies showing that psilocybin therapy was effective for depression and treatment-resistant depression in individuals with much longer durations of illness and often multiple regimens of antidepressants. By contrast, participants in this study had no prepandemic mental health history other than moderate to severe symptoms of depression, burnout, and PTSD. The efficacy of psilocybin in this study is notable given that 100% of the participants had previously tried counseling and more than 50% had tried an antidepressant. Their rapid and sustained response indicates that psilocybin therapy is a new paradigm of treatment for the postpandemic depressive symptoms in clinicians.”; “In this randomized clinical trial, psilocybin therapy was associated with a significant and sustained reduction in symptoms of depression experienced by physicians, APPs, and nurses after their frontline work during the COVID-19 pandemic. The results establish psilocybin therapy as a new paradigm of treatment for this postpandemic condition and add to the evidence of psilocybin therapy for depression. Further research is warranted to assess the role of psilocybin therapy in meeting the well-being challenges faced by clinicians in the workplace.” | 2, 8–9, 9, 10 | NA | | Vague | |  |
| Back et al., 2024 | Serotonergic System: Neuroplasticity (General) | Discussion | “Biologically, psilocybin is a partial agonist of the 5-HT2A receptor, with downstream effects of inducing neuroplasticity, which likely underlies these benefits.” | 9 | Slightly Specific | | NA | |  |
| Back et al., 2024 | Therapeutic Intervention (PAT): Emphasis on Change, Insight, Facilitation of Insight-Related Behavioral Change, and Acute Emotional Non-Avoidance | Discussion | “Therapeutically, psilocybin in this study was administered within the context of preparation, medication, and integration sessions by specially trained clinicians. These facilitators used session-specific protocols that emphasized the possibility of change, allowing emotions typically avoided, in-the-moment unfolding of the medication session, insights emerging from that experience, and cultivation of practices to bring those insights into daily life. The statistically significant difference between the psilocybin and niacin arms, both of which had the same counseling, attests to how much psilocybin added to the therapeutic intervention.” | 9 | NA | | Slightly Specific | |  |
| Back et al., 2024 | Therapeutic Intervention (PAT): Acute Emotional Non-Avoidance, Perspective, and Self-Efficacy |  | “Although the trial did not include an arm of psilocybin without therapy, the issues that emerged suggest a complex psychological landscape that would be difficult for any individual to navigate alone. Clinicians described a sense of betrayal by health systems, leaders, and colleagues; guilt from feeling that they had not been able to do enough; and grief from witnessing innumerable deaths and suffering. The question that repeatedly came up, in different forms, was “Do I matter?” What psilocybin-assisted therapy did, when it was successful in this trial, was enable participants to take some time amid the urgency of their professional and personal lives to feel all of their feelings, find some perspective on their recent past, and come to terms with what they were unable to do—and what they were able to accomplish—for patients, families, colleagues, and society.” | 9 | NA | | Slightly Specific | |  |
| Back et al., 2024 | Post-Psilocybin Behavioral Change: Resource Utilization, Self-Care, Meaning Making, and Career Changes | Discussion | “Following their experience, many participants were able to locate their own resources, give themselves permission to take care of themselves, and start to reconstruct the meaning that their work still holds. While most participants intentionally made major changes to their clinical work during their participation in the trial, none left health care altogether.” | 9 | NA | | Slightly Specific | |  |
| Carhart-Harris et al., 2021 | Neuroplasticity: Structural and Functional | Discussion | "In a study in which various psychedelic compounds were administered to rats, the compounds were shown to increase dendritic arbor complexity, promote dendritic spine growth, and stimulate synapse formation in the rat cortex, mediated by serotonin 5-HT2A receptor agonism, all of which are forms of neuronal plasticity that may be related to the principle that responses to psychedelics are especially dependent on contextual conditions." | 1410 | Highly Specific | | NA | |  |
| Carhart-Harris et al., 2021 | Neuroplasticity Interaction*Set and Setting Interaction | Discussion | "Psychedelic agents have been shown to enhance suggestibility, and their psychological effects are assumed to be context dependent. In other words, the content and subjective quality of the psychedelic experience is influenced by a person’s memories, perceptions, and degree to which the environment is supportive at the time of administration of the agent. In a study in which various psychedelic compounds were administered to rats, the compounds were shown to increase dendritic arbor complexity, promote dendritic spine growth, and stimulate synapse formation in the rat cortex, mediated by serotonin 5-HT2A receptor agonism, all of which are forms of neuronal plasticity that may be related to the principle that responses to psychedelics are especially dependent on contextual conditions." | 1410 | Highly Specific | | Vague | |  |
| Davis et al., 2021 | Serotonergic + Glutamate Systems | Introduction | "The combined serotonergic and glutamatergic action of psilocybin (a classic hallucinogen) and the preliminary evidence of the antidepressant effects of psilocybin-assisted therapy..." | 482 | Vague | | NA | |  |
| Davis et al., 2021 | Therapeutic Intervention (PAT) | Introduction, Discussion | "The combined serotonergic and glutamatergic action of psilocybin (a classic hallucinogen) and the preliminary evidence of the antidepressant effects of psilocybin-assisted therapy"; "This randomized clinical trial documented the substantial rapid and enduring antidepressant effects of psilocybin-assisted therapy among patients with MDD"; "The present findings in patients with MDD are consistent with results of studies that reported on the effectiveness of psilocybin-assisted therapy in producing antidepressant effects" | 482, 486 | NA | | Vague | |  |
| Davis et al., 2021 | Pharmacological*Psychotherapeutic Interaction | Discussion | "These findings are consistent with literature that showed that combined pharmacotherapy and psychotherapy were more efficacious in the treatment of MDD than either intervention alone." | 487 | Vague | | Vague | |  |
| Davis et al., 2021 | Subjective Experiences: Mystical Experiences | Discussion | "In several studies in patients and in healthy volunteers, the intensity of mystical-type experiences reported after psilocybin sessions was associated with favorable outcomes. Furthermore, cross-sectional studies have suggested that mystical-type and psychologically insightful experiences during a psychedelic session predict positive therapeutic effects. Consistent with these previous studies, the current trial showed that psilocybin-occasioned mystical-type, personally meaningful, and insightful experiences were associated with decreases in depression at 4 weeks (eResults in Supplement 2)." | 486-487 | NA | | Slightly Specific | |  |
| Davis et al., 2021 | Decreased Negative Affect | Discussion | "Furthermore, a recent report suggested that psilocybin may decrease negative affect and the neural correlates of negative affect, which may be a mechanism underlying transdiagnostic efficacy." | 487 | NA | | Vague | |  |
| Goodwin et al., 2022 | Subjective Experiences: Unspecified | Discussion | "Acute subjective effects of psilocybin relating to the psychedelic experience were not included as adverse events in our trial, because previous studies have suggested that they may have a mediating influence on positive outcomes." | 1645 | NA | | Vague | |  |
| Marschall et al., 2022^a^ | Behavioral/Learning: Fear Extinction | Introduction | "In contrast, Cameron et al. (2019) found no effects of a 2 month dimethyltryptamine (DMT) microdosing protocol on anxiety but did find reduced immobility in the forced swim paradigm, which is considered an antidepressant-like effect, and less freezing behaviour following fear extinction training, which may reflect enhanced fear extinction." | 98 | NA | | Slightly Specific | |  |
| Marschall et al., 2022^a^ | Cognitive-Emotional: Emotion Processing | Introduction | "Next to measuring self-reported changes in mood and anxiety, we explored potential underlying mechanisms of the alleged anxiolytic and antidepressant effects: emotion processing and interoceptive awareness." | 98 | NA | | Vague | |  |
| Marschall et al., 2022^a^ | Cognitive-Emotional: Positive-Stimuli Bias | Introduction, Hypothesis | "Our inclusion of the emotional go/no-go task was based on the double-blind, placebo-controlled study of Kometer et al. (2012; n=17), who used this task to assess alterations in emotion processing under standard doses of psilocybin and found that psilocybin increased reaction time (RT) as a function of valence...Specifically, psilocybin increased RTs more for negative and neutral go stimuli compared with positive go stimuli, thereby inducing a bias to positive stimuli."; "H3: The acute and additive action of seven psilocybin microdoses on scores of the DASS-21 is mediated by increased RTs for angry, fearful and sad facial expressions." | 98 | NA | | Highly Specific | |  |
| Marschall et al., 2022^a^ | Cognitive-Perceptual: Interoceptive Awareness | Introduction | "Next to measuring self-reported changes in mood and anxiety, we explored potential underlying mechanisms of the alleged anxiolytic and antidepressant effects: emotion processing and interoceptive awareness." | 98 | NA | | Vague | |  |
| Marschall et al., 2022^a^ | Cognitive-Perceptual: Disruptions in Top-Down Processing | Introduction | "In addition, psilocybin has been shown to disrupt preattentive sensory-motor gating, which could allow for an influx of exteroceptive and interoceptive information (Vollenweider, 2001; Vollenweider et al., 2007). Such influx may lead to increased interoceptive awareness, which has been associated with awareness and regulation of emotional states (Füstös et al., 2012). A neurocognitive mechanism which may underlie the effects of psilocybin on emotion processing and interoceptive awareness can be found in the predictive processing framework (Clark, 2013). Here, psilocybin-induced hyper-activated 5-HT2a receptors in layer V pyramidal neurons decompose the categorical topdown predictions we have about exteroceptive and interoceptive stimuli into more diverse and fine-grained predictions (PinkHashkes et al., 2017). This process may in turn disrupt top-down emotion and multisensory processing." | 98 | Highly Specific | | Highly Specific | |  |
| Marschall et al., 2022^a^ | Cognitive-Perceptual: Interoceptive Awareness | Introduction | "We did not preregister our hypothesis and analysis plan for the MAIA, but we wrote that we expected an increase in interoceptive awareness during the acute effect of the psilocybin microdose compared with placebo." | 98 | NA | | Vague | |  |
| Marschall et al., 2022^a^ | Cognitive-Emotional: Emotion Processing | Discussion | "We hypothesized that psilocybin microdosing would reduce symptoms of anxiety and depression, increase the processing time needed to identify negative emotions and increase interoceptive awareness."; "The effect of repeated microdosing on emotion processing, as measured using an emotion go/no-go task, and symptoms of anxiety, depression and stress also did not differ from placebo." | 107, 109 | NA | | Slightly Specific | |  |
| Marschall et al., 2022^a^ | Cognitive-Perceptual: Interoceptive Awareness | Discussion | "We hypothesized that psilocybin microdosing would reduce symptoms of anxiety and depression, increase the processing time needed to identify negative emotions and increase interoceptive awareness."; "Our results suggest that the psilocybin microdose did not affect interoceptive awareness" | 107 | NA | | Slightly Specific | |  |
| Marschall et al., 2022^a^ | Neuroplasticity: Structural and Functional (Prefrontal Cortex) | Discussion | "The argument that effects of microdoses may also require a longer period of repeated dosing rests on two key findings: that depression and stress-related disorders are associated with neural atrophy in the prefrontal cortex (PFC; Christoffel et al., 2011) and that serotonergic psychedelics can increase structural and functional plasticity in the PFC (Ly et al., 2018; Olson, 2018), thereby potentially counteracting the neurobiological markers of these disorders. It is possible that a period of consistent microdosing which succeeds 3 weeks is required for such changes to develop and we can expect an effect on emotion processing and mood-related symptoms only after these changes have occurred." | 110 | Slightly Specific | | Vague | |  |
| Marschall et al., 2022^a^ | Neuroplasticity: Structural and Functional (Cortex) [Anti-MoA for Microdosing, MoA for Full Doses] | | "Nevertheless, Cameron et al. (2019) administered microdoses of the serotonergic psychedelic DMT to rats every third day for 7 weeks and revealed no markers of increased neural plasticity. In fact, the researchers found a decrease in dendritic spine density in PFC of female rats. Important to note is that the original association between serotonergic psychedelics and neuronal plasticity is based on the effect of a single large serotonergic psychedelic dose. Single large doses of DMT and LSD were found to promote spinogenesis, synaptogenesis and neural plasticity in cortical neuron cultures of rats 24 h after administration (Ly et al., 2018). Taken together, this evidence, although limited in its generalizability to humans, may indicate that regardless of the duration of the dosing period, psilocybin microdoses are simply not potent enough to trigger structural changes in the cortex." | 110-111 | Slightly Specific | | NA | |  |
| Rosenblat et al., 2024 | Therapeutic Intervention (PAT) | Introduction | "Previous trials have demonstrated preliminary efficacy of psilocybin-assisted psychotherapy (PAP) in major depressive disorder (MDD), treatment resistant major depression (TRD), and patients with cancer. PAP has also been evaluated in alcohol and nicotine use disorders." | 191 | NA | | Vague | |  |
| Rosenblat et al., 2024 | Therapeutic Intervention (PAT) | Introduction | "Another unanswered question is the durability of clinical benefits and the role of repeated doses of psilocybin in acute responders who later experience relapse of depression. Results to date have suggested that even one or two doses of psilocybin, accompanied by psychotherapy, may have lasting benefits for weeks to months." | 191 | NA | | Vague | |  |
| Ross et al., 2016 | Subjective Experiences: Mystical Experiences | Introduction | "It can produce highly salient spiritual/mystical states of consciousness associated with enduring (months to years) positive changes in cognition, affect, behavior, and spirituality (Doblin, 1991; Griffiths et al., 2006, 2008, 2011; Pahnke, 1963). | 1166 | NA | | Slightly Specific | |  |
| Ross et al., 2016 | Pharmacological*Psychotherapeutic Interaction | Discussion | "It is unclear from the data whether the sustained benefits in clinical outcomes were due to psilocybin alone or some interactive effect of psilocybin plus the targeted psychotherapy. Future research would be necessary to separate out the various therapeutic contributions of psilocybin versus psychotherapy."; "and improving spiritual wellbeing (e.g. through a pharmacological/psychosocial intervention) could serve as a buffer against these negative clinical outcomes." | 1176 | Vague | | Vague | |  |
| Ross et al., 2016 | Improved Spirituality | Discussion | "…and improving spiritual wellbeing (e.g. through a pharmacological/psychosocial intervention) could serve as a buffer against these negative clinical outcomes." | 1176 | NA | | Vague | |  |
| Ross et al., 2016 | Subjective Experiences: Mystical, Meaningful, etc. | Discussion | "Psilocybin experiences were reported as highly meaningful and spiritual, and associated with positive cognitive, affective, spiritual, and behavioral effects lasting weeks to months. This finding is consistent with prior research administering psilocybin to normal volunteers (Doblin, 1991; Griffiths et al., 2006, 2008, 2011; Pahnke, 1963)." | 1176 | NA | | Vague | |  |
| Ross et al., 2016 | Subjective Experiences: Challenging Experiences | Discussion | "Difficult experiences are not necessarily pathological and can be understood as part of the therapeutic process (e.g. working through cancer-related psychological or existential distress through challenging encounters or emotionally charged confrontations with cancer-related fearful imagery or symbolism) (Carbonaro et al., 2016)." | 1176 | NA | | Vague | |  |
| Ross et al., 2016 | Serotonergic System | Discussion | "There is growing evidence that the serotoninergic psychedelics produce rapid and sustained anti-depressant effects (Nichols, 2015). In two recently published open-label trials, one using a single dose of ayahuasca (Osorio et al., 2015) and the other using two doses of oral psilocybin (Carhart-Harris et al., 2016), acute and enduring anti depressant effects were reported. In addition to these two open-label trials, there are several lines of evidence supporting using 5HT2A agonists to treat depression. In considering changes at the 5HT2A receptor as a potential mechanism of action: cortical 5HT2A receptor expression is increased in postmortem samples of patients with depression who display suicidality (Mendelson, 2000; Pandey et al., 2002; Shelton et al., 2009); depressed patients with elevated pessimism display increased PFC 5HT2A receptor binding compared to control participants (Bhagwagar et al., 2006; Meyer, 2012; Meyer et al., 2003); and sustained treatment with various anti-depressants (e.g. selective serotonin reuptake inhibitors, tricyclic antidepressants) have been associated with a reduction of 5HT2A receptor density (Gomez-Gil et al., 2004; Yamauchi et al., 2006)." | 1176 | Slightly Specific | | NA | |  |
| Ross et al., 2016 | Glutamate System: BDNF Expression + Neural Plasticity (Structural and Functional) | Discussion | "The glutamate system may explain some of the anti depressant effects of psilocybin. In rodents, serotoninergic psychedelics enhance cortical glutamatergic transmission, especially in the medial PFC, and increase activation of cortical α-amino-3- hydroxy-5-methyl-4-isoxazolepropionic acid (AMPA) receptors (Aghajanian and Marek, 1997). In a trial in which rats received DOI, there was a significant increase in expression of brain derived neurotrophic factor (BDNF) mRNA in neocortical areas (Vaidya et al., 1997). Increased AMPA activation and BDNF expression as biomarkers of anti depressant effects are supported by: cortical AMPA activation is known to stimulate the expression of cortical BDNF (associated with neuronal growth, differentiation and synaptogenesis) (Hsu et al., 2015); decreased cortical BDNF is associated with major depression in humans (Duman, 2004); and cortical BDNF normalizes with anti-depressant treatment (Sen et al., 2008; Shimizu et al., 2003). Similarly, ketamine (the only other known acute and short-term sustained anti-depressant) is theorized to exert its anti-depressant effects via cortical AMPA activation (Zanos et al., 2016) and BDNF expression (Lepack et al., 2014). However, the anti-depressant effects of single-dose ketamine in patients with TRD typically last no more than several days up to 1–2 weeks, not several weeks to months (DeWilde et al., 2015)." | 1177 | Highly Specific | | NA | |  |
| Ross et al., 2016 | Network-Level Changes: Decreased mPFC Activity + Decreased within-DMN Connectivity | Discussion | "Neuroimaging research with psilocybin is beginning to suggest potential anti-depressant mechanisms of action at the level of brain structure activity and network connectivity. Task-free functional magnetic resonance imaging research in normal volunteers under the influence of psilocybin has demonstrated decreased activity in the medial PFC and decreased connectivity within the default mode network (DMN) (Carhart-Harris et al., 2012, 2014). The former is significant because depressive symptoms have been associated with increased activity in the medial PFC (Drevets et al., 2008; Farb et al., 2011) and normalization of medial PFC activity has been demonstrated with anti-depressant treatment (Deakin et al., 2008; Holtzheimer and Mayberg, 2011; Kennedy et al., 2007); and the latter because patients with major depression (compared to controls) have demonstrated increased DMN connectivity (Berman et al., 2011, Grecius et al., 2007)." | 1177 | Highly Specific | | NA | |  |
| Ross et al., 2016 | Subjective Experiences: Mystical Experiences | Discussion | "Psycho-spiritual mechanisms. Moderate-dose psilocybin occasioned mystical-type experiences in the cohort of cancer patients studied, and the intensity of the subjective mystical experience significantly mediated (e.g. suggestive of causality) clinical benefit (e.g. reduction in anxiety and depression symptoms) in the medium term (e.g. 6 weeks post-dose 1). This result matches with descriptive historical data from open-label LSD-assisted psychotherapy trials for psycho-spiritual distress associated with terminal cancer, in which the mystical experience was reported as being an integral part of the therapeutic effect (Grof and Halifax, 1977). It is further corroborated by recent open-label trials using psilocybin-assisted psychotherapy to treat tobacco addiction (Garcia-Romeu et al., 2014; Johnson et al., 2014) and alcoholism (Bogenschutz et al., 2015) showing significant correlations between the mystical experience and improved clinical outcomes. This finding suggests a potential psycho-spiritual mechanism of action: the mystical state of consciousness. The mystical experience is likely to be one of several mediators that transmit the effect of psilocybin to changes in anxiety and/or depression. Further enquiry into how particular dimensions of the mystical experience relate to reductions in anxiety and/or depression in this population and others, and what factors best predict or promote mystical experiences, is warranted."; "...The psilocybin-induced mystical experience mediated the anxiolytic and anti-depressant effects of psilocybin." | 1177 | NA | | Highly Specific | |  |
| von Rotz et al., 2023 | Serotonergic System | Introduction | "In the search for new treatments, a pilot study and two controlled clinical trials have shown that the psychotropic substance psilocybin - a preferential serotonin 1A/2A receptor agonist – in combination with psychological support, rapidly and sustainably alleviates depressive symptoms in MDD." | 2 | Vague | | NA | |  |
| von Rotz et al., 2023 | Therapeutic Intervention (PAT) | Introduction | "In the search for new treatments, a pilot study and two controlled clinical trials have shown that the psychotropic substance psilocybin - a preferential serotonin 1A/2A receptor agonist – in combination with psychological support, rapidly and sustainably alleviates depressive symptoms in MDD."; "Specifically, in an open-label feasibility study, administration of two doses of psilocybin (10 mg and 25 mg, 7 days apart) together with psychological support resulted in rapid symptom improvement in patients with treatment-resistant depression that persisted at a 6 months follow-up."; "Given that a rapid decrease in symptom severity was observed in both intervention arms, it is possible that the psychological support and the low dose of psilocybin may have contributed significantly to the therapeutic effect..."; "...(equal time spent with psychological support in both treatment conditions)..."; "...ensure appropriate psychological support during the preparation, drug, and integration sessions of the experience." | 2 | NA | | Vague | |  |
| von Rotz et al., 2023 | Therapeutic Intervention (PAT) | Discussion | "Furthermore, in the present study, psilocybin-assisted therapy appears to produce a similar rapid onset of antidepressant action" | 9 | NA | | Vague | |  |
| von Rotz et al., 2023 | Neurocognitive-Emotional: Emotion-Processing, Positive-Stimuli Bias | Discussion | "Aligning with this, recent studies demonstrated that psilocybin-assisted therapy could bias negative emotion processing to the positive through modulation of limbic structures and associated neural networks." | 9 | Slightly Specific | | Vague | |  |
| von Rotz et al., 2023 | Subjective Experiences: Unspecified [Anti-MoA] | Discussion | "As shown in Fig. 3, most of the participants in the psilocybin group experienced substantial treatment induced subjective effects, while most participants in the placebo group reported only minor subjective effects. The intensity of the psilocybin-induced subjective effects assessed by the ASC global score (for subscales, see Supplemental Fig. S3) did not correlate with the reduction in depressive symptomatology at 2-week posttreatment. Hence, the present results do not corroborate the assumption that the degree of the drug induced subjective effects predicts the beneficial outcome in MDD. To what extent additional non-pharmacological factors (i.e., psychological aspects) have shaped subjective drug effects and may contribute to the beneficial therapeutic outcome remains to be further investigated. In fact, the necessity of the subjective experience of psychedelics for the enduring therapeutic effects has recently been challenged and is yet debated. Thus, these present findings must be interpreted with caution. Further mechanistic studies including more specific measures of cognitive and emotional processing may help to clarify this important issue. " | 10 | NA | | Slightly Specific | |  |
| von Rotz et al., 2023 | Subjective Experiences: Unspecified [Anti-MoA] | Discussion | "Treatment response was not associated with the intensity of subjective drug effects highlighting the need for further investigations into the psychological and neuronal mechanisms of action of psychedelic substances." | | 10 | NA | | Vague | |
| *Note.* Numerical in-text citations were removed from quotes to promote ease of readings. Intro. = introduction. Num. = number. PAT = psychedelic-assisted therapy. MoA = mechanism of action. ^a^Marschall et al., 2022 studied microdosing, which has several implications for a mechanism-of-action analysis: 1) mechanisms of action may be different between microdosing and full-dose psychedelic administration or PAT; 2) it may be easier to study certain mechanisms of action in the context of microdosing as microdosing trials do not face all of the same blinding and expectancy confounds as clinical trials of full doses of psychedelics. Raison et al., 2023 did not offer any discussion of MoAs that qualified for the present synthesis. | | | | | | | | | |
