# Supplementary material for: Incremental efficacy systematic review and meta-analysis of psilocybin-for-depression RCTs
Source: Psychopharmacology (Berl). 2025 Apr 23;242(10):2139–57. doi: 10.1007/s00213-025-06788-w (PMC12449434; doi:10.1007/s00213-025-06788-w)
Supplement: Supplementary file 6 — Supplementary file6 (DOCX 36 KB) [file 213_2025_6788_MOESM6_ESM.docx]

| Study Name | Subgroups Compared | Psilocybin Dosage | Psilocybin Dose Number | Control type | Timepoint | Measure | Hedges' G | Standard Error | 95% CI |
| --- | --- | --- | --- | --- | --- | --- | --- | --- | --- |
| Back et al. (2024) | Psilocybin vs. Niacin | 25mg | 1 | 100mg Niacin | 28 | MADRS | 1.34 | 0.395 | (0.566, 2.114), |
| Carhart-Harris et al., 2021 | Psilocybin vs. Escitalopram | 25mg | 2 | 1mg Psilocybin and Escitalopram | 14.00 | BDI-1A | 0.721 | 0.272 | (0.187, 1.255) |
| ^Carhart- Harris et al., 2021 | Psilocybin vs. Escitalopram | 25mg | 2 | 1mg Psilocybin and Escitalopram | 28.00 | BDI-1A | 0.309 | 0.259 | (0.816, -0.198) |
| Carhart-Harris et al., 2021 | Psilocybin vs. Escitalopram | 25mg | 2 | 1mg Psilocybin and Escitalopram | 42.00 | BDI-1A | 0.332 | 0.259 | (-0.175, 0.839) |
| Carhart-Harris et al., 2021 | Psilocybin vs. Escitalopram | 25mg | 2 | 1mg Psilocybin and Escitalopram | 42.00 | HAM-D-17 | 0.185 | 0.262 | (-0.329, 0.698) |
| Carhart-Harris et al., 2021 | Psilocybin vs. Escitalopram | 25mg | 2 | 1mg Psilocybin and Escitalopram | 42.00 | MADRS | 0.151 | 0.262 | (-0.362, 0.664) |
| Carhart-Harris et al., 2021 | Psilocybin vs. Escitalopram | 25mg | 2 | 1mg Psilocybin and Escitalopram | 3.00 | QIDS-SR-1 | 0.086 | 0.268 | (-0.440, 0.612) |
| Carhart-Harris et al., 2021 | Psilocybin vs. Escitalopram | 25mg | 2 | 1mg Psilocybin and Escitalopram | 22.00 | QIDS-SR-1 | -0.025 | 0.274 | (-0.562, 0.512) |
| Carhart-Harris et al., 2021 | Psilocybin vs. Escitalopram | 25mg | 2 | 1mg Psilocybin and Escitalopram | 7.00 | QIDS-SR-1 | 0.074 | 0.267 | (-0.450, 0.597) |
| ^Carhart- Harris et al., 2021 | Psilocybin vs. Escitalopram | 25mg | 2 | 1mg Psilocybin and Escitalopram | 14.00 | QIDS-SR-1 | 0.201 | 0.258 | (0.706, -0.304) |
| ^Carhart- Harris et al., 2021 | Psilocybin vs. Escitalopram | 25mg | 2 | 1mg Psilocybin and Escitalopram | 21.00 | QIDS-SR-1 | 0.305 | 0.259 | (0.812, -0.201) |
| ^Carhart- Harris et al., 2021 | Psilocybin vs. Escitalopram | 25mg | 2 | 1mg Psilocybin and Escitalopram | 28.00 | QIDS-SR-1 | 0.412 | 0.260 | (0.921, -0.097) |
| ^Carhart- Harris et al., 2021 | Psilocybin vs. Escitalopram | 25mg | 2 | 1mg Psilocybin and Escitalopram | 35.00 | QIDS-SR-1 | 0.309 | 0.259 | (0.816, -0.198) |
| Carhart-Harris et al., 2021 | Psilocybin vs. Escitalopram | 25mg | 2 | 1mg Psilocybin and Escitalopram | 42.00 | QIDS-SR-1 | 0.041 | 0.257 | (-0.463, 0.544) |
| ^Carhart- Harris et al., 2021 | Psilocybin vs. Escitalopram | 25mg | 2 | 1mg Psilocybin and Escitalopram | 49.00 | QIDS-SR-1 | 0.323 | 0.259 | (0.830, -0.184) |
| Davis et al., 2021 | Psilocybin vs. Waitlist control | 20 mg/70kg and 30 mg/70kg (28.6mg) | 2 | Waitlist Control | 35.00 | BDI-II | 2.620 | 0.547 | (1.548, 3.693) |
| Davis et al., 2021 | Psilocybin vs. Waitlist control | 20 mg/70kg and 30 mg/70kg (28.6mg) | 2 | Waitlist Control | 56.00 | BDI-II | 3.160 | 0.604 | (1.977, 4.344) |
| Davis et al., 2021 | Psilocybin vs. Waitlist control | 20 mg/70kg and 30 mg/70kg (28.6mg) | 2 | Waitlist Control | 35.00 | GRID-HAM | 2.450 | 0.531 | (1.410, 3.490) |
| Davis et al., 2021 | Psilocybin vs. Waitlist control | 20 mg/70kg and 30 mg/70kg (28.6mg) | 2 | Waitlist Control | 56.00 | GRID-HAM | 2.547 | 0.540 | (1.488, 3.605) |
| Davis et al., 2021 | Psilocybin vs. Waitlist control | 20 mg/70kg and 30 mg/70kg (28.6mg) | 2 | Waitlist Control | 35.00 | QIDS-SR | 4.848 | 0.804 | (3.272, 6.423) |
| Davis et al., 2021 | Psilocybin vs. Waitlist control | 20 mg/70kg and 30 mg/70kg (28.6mg) | 2 | Waitlist Control | 56.00 | QIDS-SR | 2.839 | 0.569 | (1.722, 3.955) |
| ^Goodwin et al. | Low dose psilocybin | 10mg | 1 | 10mg psilocybin | 3.00 | MADRS | 0.239 | 0.161 | (0.555, -0.076) |
| ^Goodwin et al. | Low dose psilocybin | 10mg | 1 | 10mg psilocybin | 8.00 | MADRS | 0.168 | 0.161 | (0.483, -0.147) |
| Goodwin et al. | Low dose psilocybin | 10mg | 1 | 10mg psilocybin | 22.00 | MADRS | 0.190 | 0.161 | (0.505, -0.126) |
| ^Goodwin et al. | Low dose psilocybin | 10mg | 1 | 10mg psilocybin | 55.00 | MADRS | 0.028 | 0.160 | (0.342, -0.287) |
| ^Goodwin et al. | Low dose psilocybin | 10mg | 1 | 10mg psilocybin | 64.00 | MADRS | 0.055 | 0.160 | (0.370, -0.259) |
| ^Goodwin et al. | Low dose psilocybin | 10mg | 1 | 10mg psilocybin | 85.00 | MADRS | 0.020 | 0.160 | (0.335, -0.294) |
| ^Goodwin et al. | High dose psilocybin | 25mg | 1 | 25mg psilocybin | 3.00 | MADRS | 0.492 | 0.161 | (0.807, 0.177) |
| ^Goodwin et al. | High dose psilocybin | 25mg | 1 | 25mg psilocybin | 8.00 | MADRS | 0.542 | 0.161 | (0.858, 0.226) |
| Goodwin et al. | High dose psilocybin | 25mg | 1 | 25mg psilocybin | 22.00 | MADRS | 0.429 | 0.160 | (0.742, 0.115) |
| ^Goodwin et al. | High dose psilocybin | 25mg | 1 | 25mg psilocybin | 55.00 | MADRS | 0.385 | 0.160 | (0.699, 0.072) |
| ^Goodwin et al. | High dose psilocybin | 25mg | 1 | 25mg psilocybin | 64.00 | MADRS | 0.288 | 0.159 | (0.600, -0.024) |
| ^Goodwin et al. | High dose psilocybin | 25mg | 1 | 25mg psilocybin | 85.00 | MADRS | 0.242 | 0.159 | (0.554, -0.069) |
| Marschall et al., 2022 | Psilocybin vs. Placebo | 0.7g Dried Golden Truffles | 7 | Placebo | 21.00 | DASS-21 | 0.051 | 0.309 | (0.656, -0.554) |
| Raison et al., 2023 | Psilocybin vs. Niacin | 25mg | 1 | 100mg Niacin | 2.00 | MADRS | 0.065 | 0.195 | (0.446, -0.317) |
| Raison et al., 2023 | Psilocybin vs. Niacin | 25mg | 1 | 100mg Niacin | 8.00 | MADRS | 1.086 | 0.209 | (0.677, 1.496) |
| Raison et al., 2023 | Psilocybin vs. Niacin | 25mg | 1 | 100mg Niacin | 15.00 | MADRS | 0.911 | 0.205 | (0.510, 1.312) |
| Raison et al., 2023 | Psilocybin vs. Niacin | 25mg | 1 | 100mg Niacin | 29.00 | MADRS | 1.077 | 0.209 | (0.668, 1.486) |
| Raison et al., 2023 | Psilocybin vs. Niacin | 25mg | 1 | 100mg Niacin | 43.00 | MADRS | 0.907 | 0.205 | (0.506, 1.309) |
| Raison et al., 2023 | Psilocybin vs. Niacin | 25mg | 1 | 100mg Niacin | 43.00 | Oxford | 0.813 | 0.203 | (0.416, 1.210) |
| Raison et al., 2023 | Psilocybin vs. Niacin | 25mg | 1 | 100mg Niacin | 1.00 | Symptoms | 0.133 | 0.195 | (-0.249, 0.515) |
| Raison et al., 2023 | Psilocybin vs. Niacin | 25mg | 1 | 100mg Niacin | 8.00 | Symptoms | 0.879 | 0.204 | (0.479, 1.278) |
| Raison et al., 2023 | Psilocybin vs. Niacin | 25mg | 1 | 100mg Niacin | 43.00 | Symptoms | 0.704 | 0.201 | (0.311, 1.098) |
| ^Rosenblat et al., 2024 | Psilocybin | 25mg | 1 | Waitlist Control | 1.00 | MADRS | 1.872 | 0.430 | (2.716, 1.029) |
| ^Rosenblat et al., 2024 | Psilocybin | 25mg | 1 | Waitlist Control | 7.00 | MADRS | 1.064 | 0.382 | (1.812, 0.316) |
| Rosenblat et al., 2024 | Psilocybin | 25mg | 1 | Waitlist Control | 14.00 | MADRS | 1.067 | 0.382 | (0.319, 1.815) |
| ^Ross et al., 2016 | Psilocybin vs. Niacin | 0.3mg/kg | 1 | 250mg Niacin | 2.00 | BDI | 0.264 | 0.363 | (0.975, -0.447) |
| ^Ross et al., 2016 | Psilocybin vs. Niacin | 0.3mg/kg | 1 | 250mg Niacin | 15.00 | BDI | 0.129 | 0.367 | (0.849, -0.591) |
| ^Ross et al., 2016 | Psilocybin vs. Niacin | 0.3mg/kg | 1 | 250mg Niacin | 43.00 | BDI | 0.233 | 0.368 | (0.955, -0.489) |
| ^Ross et al., 2016 | Psilocybin vs. Niacin | 0.3mg/kg | 1 | 250mg Niacin | 50.00 | BDI | 0.031 | 0.381 | (0.778, -0.715) |
| ^Ross et al., 2016 | Psilocybin vs. Niacin | 0.3mg/kg | 1 | 250mg Niacin | 2.00 | HADS-D | 0.597 | 0.370 | (1.321, -0.128) |
| ^Ross et al., 2016 | Psilocybin vs. Niacin | 0.3mg/kg | 1 | 250mg Niacin | 15.00 | HADS-D | 0.484 | 0.373 | (1.214, -0.246) |
| ^Ross et al., 2016 | Psilocybin vs. Niacin | 0.3mg/kg | 1 | 250mg Niacin | 43.00 | HADS-D | 0.679 | 0.378 | (1.420, -0.062) |
| ^Ross et al., 2016 | Psilocybin vs. Niacin | 0.3mg/kg | 1 | 250mg Niacin | 50.00 | HADS-D | 0.337 | 0.384 | (1.090, -0.415) |
| ^Von Rotz et al., 2023 | Psilocybin | 0.215mg/kg | 1 | Placebo (Mannitol) | 1.00 | BDI | 0.973 | 0.289 | (1.540, 0.406) |
| ^Von Rotz et al., 2023 | Psilocybin | 0.215mg/kg | 1 | Placebo (Mannitol) | 3.00 | BDI | 1.105 | 0.294 | (1.681, 0.529) |
| ^Von Rotz et al., 2023 | Psilocybin | 0.215mg/kg | 1 | Placebo (Mannitol) | 9.00 | BDI | 0.754 | 0.283 | (1.309, 0.199) |
| ^Von Rotz et al., 2023 | Psilocybin | 0.215mg/kg | 1 | Placebo (Mannitol) | 15.00 | BDI | 0.760 | 0.283 | (1.315, 0.205) |
| Von Rotz et al., 2023 | Psilocybin | 0.215mg/kg | 1 | Placebo (Mannitol) | 1.00 | MADRS | 0.943 | 0.288 | (1.508, 0.378) |
| Von Rotz et al., 2023 | Psilocybin | 0.215mg/kg | 1 | Placebo (Mannitol) | 3.00 | MADRS | 0.999 | 0.290 | (1.568, 0.430) |
| Von Rotz et al., 2023 | Psilocybin | 0.215mg/kg | 1 | Placebo (Mannitol) | 9.00 | MADRS | 0.672 | 0.281 | (1.223, 0.121) |
| Von Rotz et al., 2023 | Psilocybin | 0.215mg/kg | 1 | Placebo (Mannitol) | 15.00 | MADRS | 0.815 | 0.285 | (1.373, 0.258) |
| Von Rotz et al., 2023 | Psilocybin | 0.215mg/kg | 1 | Placebo (Mannitol) | 15.00 | SCL-90-R | 0.701 | 0.287 | (1.264, 0.139) |

*Note:* For Ross et al., 2016 we are using 1-day pre psilocybin administration as the baseline, where results come from their NCT. ^Denotes estimates were extracted using the Web Plot Digitizer tool (version 4; Automeris LLC., 2024). Please also note that each of these effects are presented as independent in supplementary file 16. These effects are displayed for descriptive purposes only. If these effect sizes are used for future meta-analyses, effects need to be re-calculated to account for violations of independence associated with multiple effects coming from identical subgroups/studies.
