# Supplementary material for: Incremental efficacy systematic review and meta-analysis of psilocybin-for-depression RCTs
Source: Psychopharmacology (Berl). 2025 Apr 23;242(10):2139–57. doi: 10.1007/s00213-025-06788-w (PMC12449434; doi:10.1007/s00213-025-06788-w)
Supplement: Supplementary file 7 — Supplementary file7 (DOCX 16 KB) [file 213_2025_6788_MOESM7_ESM.docx]

**Supplementary File 15**

Email Tracking Sent to Corresponding Authors

| Study Name | Reason for Email | Number of Emails Sent | Heard from? |
| --- | --- | --- | --- |
| Back et al. 2024 | Data Requested | 1 | No |
| Carhart-Harris et al., 2021 | Data Requested | 3 | No |
| Davis et al., 2021 | N/A | 0 | N/A |
| Goodwin et al., 2022 | Data Requested | 1 | Yes |
| Grob et al. 2011 | Data Requested and Study Clarification | 1 | No |
| Marschall et al., 2022 | N/A | 0 | N/A |
| Raison et al., 2023 | Data Requested | 2 | Yes |
| Rosenblat et al., 2024 | Data Requested | 3 | No |
| Ross et al., 2016 | Data Requested and Study Clarification | 5 | Yes* |
| von Rotz et al., 2023 | Data Requested and Study Clarification | 4 | No |

*Did eventually respond to email. Indicated that clinicaltrial.gov displayed outcome data that was inaccurate and provided a SAS output for one outcome measure.
